# Potential Applications of Nanoparticles in Improving the Outcome of Lung Cancer Treatment

**DOI:** 10.3390/genes14071370

**Published:** 2023-06-28

**Authors:** Agnishwar Girigoswami, Koyeli Girigoswami

**Affiliations:** Medical Bionanotechnology, Faculty of Allied Health Sciences, Chettinad Hospital and Research Institute, Chettinad Academy of Research and Education, Chettinad Health City, Kelambakkam, Chennai 603103, India

**Keywords:** lung cancer, chemotherapy, radiation therapy, nano sensitizers, radiation protection by nanoparticles

## Abstract

Lung cancer is managed using conventional therapies, including chemotherapy, radiation therapy, or a combination of both. Each of these therapies has its own limitations, such as the indiscriminate killing of normal as well as cancer cells, the solubility of the chemotherapeutic drugs, rapid clearance of the drugs from circulation before reaching the tumor site, the resistance of cancer cells to radiation, and over-sensitization of normal cells to radiation. Other treatment modalities include gene therapy, immunological checkpoint inhibitors, drug repurposing, and in situ cryo-immune engineering (ICIE) strategy. Nanotechnology has come to the rescue to overcome many shortfalls of conventional therapies. Some of the nano-formulated chemotherapeutic drugs, as well as nanoparticles and nanostructures with surface modifications, have been used for effective cancer cell killing and radio sensitization, respectively. Nano-enabled drug delivery systems act as cargo to deliver the sensitizer molecules specifically to the tumor cells, thereby enabling the radiation therapy to be more effective. In this review, we have discussed the different conventional chemotherapies and radiation therapies used for inhibiting lung cancer. We have also discussed the improvement in chemotherapy and radiation sensitization using nanoparticles.

## 1. Introduction

**Figure 1:**
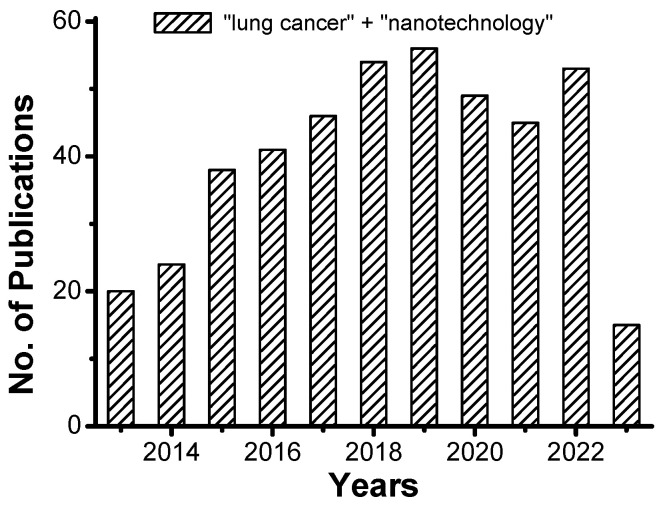
The total number of original research publications on the application of nanotechnology in lung cancer.

Men and women die from lung cancer at a higher rate than any other cancer in the US and around the world. Nearly 84% of all lung cancers belong to non-small-cell lung cancers (NSCLC), while 13% are small-cell lung cancers (SCLC). However, lung cancer screening has expanded, radiation techniques have improved, and treatment advances have changed the prognosis for NSCLC markedly in the past decade. The reported decline in NSCLC mortality is likely a result of these changes. According to a recent study, men’s lung cancer incidence decreased by 3%, and women’s by 1% during the last decade [1]. Over the past decade, increased screening has likely contributed to a rise in stage I NSCLC at diagnosis. As a result of earlier diagnosis of NSCLC and more effective treatment, the prevalence of NSCLC has increased, and five-year survival has improved. Undertreatment may be to blame for the low five-year survival rate among patients aged 65 and older with stage IV NSCLC despite the availability of effective treatments [1,2]. Antihypertensives, anti-hyperlipidemia drugs, anti-inflammatory drugs, anti-diabetics, and antimicrobials are examples of potential candidate drugs that can be repurposed for treating NSCLC [2]. There has been a tremendous leap forward in the field of nanotechnology in recent times, highlighting its applications in the field of targeted drug delivery, imaging, and especially in cancer theranostics [3,4]. Nanobiotechnology-based drug delivery systems are used as drug delivery systems to target tumor cells due to their properties like sustainable biocompatibility, biodistribution, and active targeting. Control of the matter at the nanoscale plays a significant role in these technologies. Nanoparticles are used explicitly due to their properties like confined size, magnetic and optoelectronic properties, and atomic structure. They can also be used for site-specific targeting due to all these properties; nanobiotechnology has advanced in the treatment of cancer [5]. Nanoparticles like liposomes, nanofiber, nanoshells, micelles, dendrimers, carbon nanotubes, and quantum dots are used as nanocarriers that help in carrying the anticancer drug and delivering it to a specific site. These formulations protect the drug from degradation and improve its efficiency and stability. Therefore, they can be used for the targeted therapy of lung cancer [6]. The treatment of cancer with immunotherapy, in which the immune system recognizes and attacks tumors, is a promising approach. The tumor microenvironment (TME) has an immunosuppressive (i.e., immunologically cold) nature, which significantly limits the immune system’s effectiveness. A cryo-immune engineering (ICIE) strategy has been developed for converting a “cold” TME into a “hot” one. Not only in primary tumors with cryosurgery but also in distant tumors without freezing, the ratio of CD8+ cytotoxic T cells to immunosuppressive regulatory T cells increases by more than 100 times after ICIE treatment. The anticancer drug and PD-L1 silencing siRNA are rapidly released into the cytosol following cryosurgery using cold-responsive nanoparticles that target tumors and cause “frostbite” of tumors [7]. Although ICIE therapy has been used recently for breast cancer models in female mice, it can be extrapolated for lung cancer models in the future. A wider holistic approach is necessary to study more about the recent treatment strategies for lung cancer and how they overcome the shortfalls of the conventional treatment modalities. This review is warranted for an updated understanding of improved nanotechnology-based theranostic approaches in the management of lung cancer. The number of original research publications published so far during the past 10 years on the use of various nanotechnology applications for lung cancer treatment and therapies is represented in Figure 1. The figure was prepared by using the search engine “Google Scholar” and “PubMed” using appropriate keywords (“lung cancer” + “nanotechnology”).

## 2. Chemotherapy for Treatment of Lung Cancer

**Figure 2:**
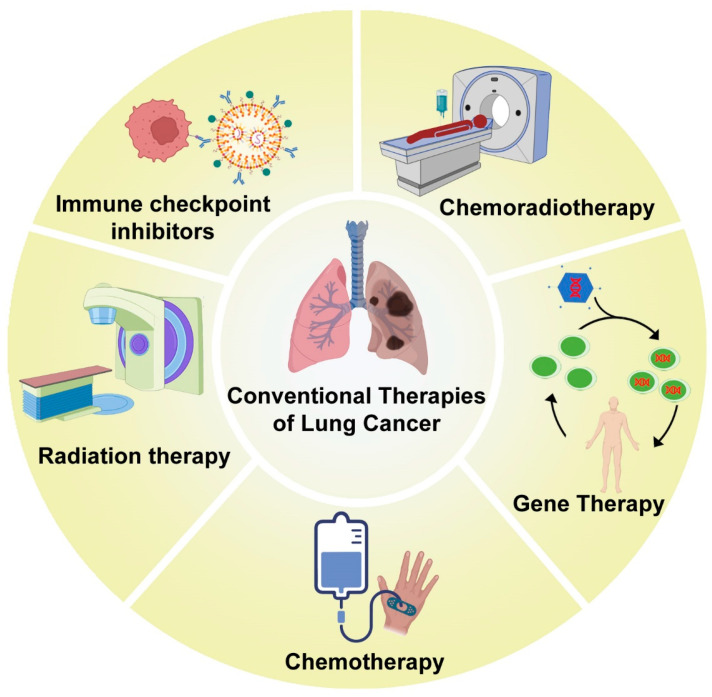
The conventional lung cancer treatment modalities.

The conventional therapies for the management of lung cancer include chemotherapy and radiation therapy, as well as a combination of both. The different kinds of traditional treatment modalities for lung cancer management are shown in Figure 2.

Among the chemotherapeutic drugs, the modes of action encompass mitotic inhibitors, alkylating agents, topoisomerase inhibitors, antimetabolites, tubulin-binding agents, etc. [8]. Forde et al. performed an open-label, phase 3 trial by randomly assigning patients with resectable NSCLC stages IB to IIIA. They received (i) nivolumab plus platinum-based chemotherapy or (ii) only platinum-based chemotherapy, followed by surgical resection. By blinded independent review, the primary endpoints were the number of event-free days and the percentage of pathologically complete responses (0% viable tumor detected in resected lymph nodes and lung). As a secondary outcome, overall survival was important, and each patient was assessed for safety. Patients with resectable NSCLC treated with neoadjuvant nivolumab and chemotherapy achieved a higher rate of complete pathological responses than those treated with chemotherapy alone. Neoadjuvant chemotherapy with nivolumab did not increase the incidence of adverse events or impair the feasibility of surgery [9]. A study conducted by Ares et al. examined whether the conjugation of two chemotherapy cycles to nivolumab plus ipilimumab would enhance the clinical benefit further in 1152 patients. A combination of nivolumab plus ipilimumab plus two cycles of chemotherapy significantly improved overall survival compared to chemotherapy alone. Based on these results, this regimen is likely to be an effective first-line therapy for advanced NSCLC patients [10]. According to another study, patients with advanced squamous non-small-cell lung cancer (sq-NSCLC) who received tislelizumab along with chemotherapy had improved PFS. An assessment was performed by an independent review committee (IRC) regarding the PFS. Secondary endpoints were OS, IRC-assessed objective response rate (ORR), investigator-assessed PFS, and IRC-assessed duration of response, along with adverse events (AEs). According to the results of this phase 3 randomized clinical study, patients with advanced sq-NSCLC treated with tislelizumab had significantly longer PFS, higher ORRs, and a more manageable safety/tolerability profile, regardless of the expression of PD-L1 [11].

### 2.1. Chemoimmunotherapy

At an advanced level, immunotherapy is also combined with chemotherapy to yield a superior result and improve the effective lifespan of the patient. In NSCLC patients with previously treated cancer, atezolizumab (a monoclonal antibody against programmed cell death ligand (PD-L1)) improved overall survival by restoring anticancer immunity. It also demonstrated clinical benefit as a first-line treatment when combined with chemotherapy. For non-squamous NSCLC patients, this study compared the effectiveness and safety of atezolizumab plus chemotherapy with chemotherapy alone. A combined 723 patients from eight countries participated in this study, and it showed that atezolizumab plus chemotherapy improved progression-free survival (PFS) and overall survival (OS) significantly versus chemotherapy when used as first-line of treatment for patients with stage IV non-squamous NSCLC without mutations of EGFR or ALK. Atezolizumab was effective in treating metastatic NSCLC in combination with platinum-based chemotherapy, as supported by this study [12]. Based on the effectiveness of immune checkpoint inhibitors in metastatic NSCLC, researchers planned a trial to assess the efficacy of atezolizumab combined with carboplatin and nab-paclitaxel as neoadjuvant therapy before surgical removal of metastatic NSCLC. Three American hospitals participated in this open-label, single-arm, multicenter, phase 2 trial. It was concluded that nab-paclitaxel and atezolizumab plus carboplatin could be used as the neoadjuvant regimen in resectable NSCLC, showing a high pathological response rate and controllable toxic effects that did not impair the surgical resection in most patients [13].

A checkpoint inhibitor targeting programmed cell death 1 (PD-1) or its ligand (PD-L1) exerted clinical activity in patients with metastatic NSCLC. An open-label, phase 3 randomized clinical trial (MYSTIC) was executed throughout 17 countries at 203 centers for cancer treatment to compare durvalumab, with or without tremelimumab, with chemotherapy as the first-line treatment for metastatic NSCLC. Patients with 25% of tumor cells expressing PD-L1 in the phase 3 MYSTIC trial failed to achieve the primary endpoints showing improved OS after treatment with durvalumab in comparison with chemotherapy, or improved PFS or OS with durvalumab and tremelimumab, both compared to chemotherapy. The combined use of durvalumab plus tremelimumab with a bTMB threshold of 20 mutations per megabase provided an ideal OS benefit [14]. Another study, conducted on 361 and 358 patients, compared nivolumab (NIVO) plus ipilimumab (IPI) plus two cycles of platinum-doublet chemotherapy (chemo) to only chemo in stage IV/recurrent NSCLC in 1 L stage IV. With NSCLC-optimized IPI + NIVO + a limited amount of chemo in comparison to chemotherapy (4 cycles) in 1 L advanced NSCLC, there was a significant OS enhancement. No new safety signals were reported during this study [15]. The same group of scientists reported the first five-year follow-up data from any phase III trial involving immunotherapy as part of a first-line treatment for NSCLC. As part of a randomized controlled trial (RCT), pembrolizumab was compared with platinum-based chemotherapeutic drugs for previously untreated NSCLC diagnosed with at least 50% tumor proportion score in terms of PD-L1 and a lack of sensitizing changes in EGFR or ALK. The patients were assigned randomly (1:1) to receive pembrolizumab (200 mg once every 3 weeks for 35 cycles) or platinum-based chemotherapy. Pembrolizumab could be given to chemotherapy patients with progressive disease, and 305 patients participated in this study. When used for first-line therapy in cases of metastatic NSCLC with at least a 50% PD-L1 tumor proportion score, pembrolizumab provided a durable, clinically meaningful OS benefit over chemotherapy [16].

A study by Provencio et al. examined the antitumor activity of, as well as safety issues for, neoadjuvant chemoimmunotherapy applied to stage IIIA resectable NSCLC. In this study, neoadjuvant nivolumab was added to platinum-based chemotherapy, given to resectable stage IIIA NSCLC patients. Chemoimmunotherapy as a neoadjuvant treatment for locally advanced lung cancer may change perceptions of lung cancer as a potentially lethal disease into a curable one [17].

### 2.2. Tyrosine Kinase Inhibitor with Chemotherapy

An epidermal growth factor receptor (EGFR)—directed oral inhibitor of tyrosine kinase was the standard first-line treatment in cases of advanced NSCLC. Oral tyrosine kinase inhibitors can be complemented with chemotherapy using pemetrexed and carboplatin. In a phase III randomized trial, Noronha et al. evaluated first-line palliative therapy in advanced NSCLC patients with EGFR-sensitizing mutations and monitored a performance status increment from 0 to 2. According to the results of the study with 350 patients, adding chemotherapy with gefitinib did significantly prolong PFS and OS, but nonetheless increased toxicity [18]. Treatment of advanced NSCLC with mutations in EGFR with a tyrosine kinase inhibitor and cytotoxic chemotherapy was highly effective. A total of 345 patients newly diagnosed with metastatic NSCLC having EGFR mutations were randomly assigned to receive either gefitinib alone or gefitinib along with carboplatin and pemetrexed. According to a hierarchical sequential testing method, progression-free survival (PFS, PFS2) as well as OS were analyzed sequentially. Quality of life, safety, and objective response rate (ORR) were secondary endpoints. Compared to gefitinib alone, the combination of gefitinib with pemetrexed plus carboplatin showed improvement in PFS in untreated advanced NSCLC patients with mutations in EGFR, but further study is needed to determine its OS benefit [19]. Previous studies have investigated whether chemotherapy using single or multiple chemotherapeutic drugs or a combination of immunotherapy with chemotherapy could improve the progression of lung cancer. But it was evident that these treatment modalities had a few limitations, such as toxicity, quality of survival, etc.

## 3. Lung Cancer Management by Radiation Therapy

The American Society for Radiation Oncology has set clear radiation guidelines for the management of different types and stages of lung cancer [20]. SCLC has recently been challenged in a number of sentinel phase III randomized trials. Both limited-stage (LS) and extensive-stage (ES) SCLC can benefit from thoracic radiotherapy and prophylactic cranial irradiation (PCI). Initially, during the treatment course for LS-SCLC, definitive thoracic RT should be administered once or twice daily. If a patient has positive margins or nodal metastases after surgical resection, adjuvant RT is conditionally recommended. Also, involved field RT used as an advanced treatment modality was recommended strongly for post-chemotherapy volumes. Stereotactic body radiation therapy (SBRT) or conventional fractionation is strongly recommended for patients with stage I or II node-negative cancer, and chemotherapy should be administered before or after SBRT. Patients with stage II or III LS-SCLC who responded to chemoradiation are strongly recommended to undergo PCI; those at high risk of neurocognitive toxicity should be included in the decision. It is strongly recommended to consult a radiation oncologist regarding PCI vs. magnetic resonance surveillance in ES-SCLC. A conditional recommendation is also made for thoracic radiotherapy usage in patients with ES-SCLC following chemotherapy. As in LS-SCLC, RT plays a crucial role in ES-SCLC as well. In SCLC, these guidelines provide guidance on the best clinical practices for local therapy [21].

### 3.1. Stereotactic Body Radiation Therapy

Radiation therapy has been used for controlling the lung cancerous tumors in 77% of patients [22]. A randomized trial for lung SBRT comparing 34 Gy in one fraction with 48 Gy in four fractions was conducted for presenting the long-term outcome of RTOG 0915/NCCTG N0927. A phase 2 multicenter study involved medically inoperable patients with metastatic peripheral T1 or T2 N0M0 NSCLC. The primary endpoint was one-year toxicity, with secondary endpoints including failure and survival. Neither arm showed a significant increase in late-appearing toxicity. The primary tumor control rates at 5 years were similar between the two arms. In a study with 84 patients, a median time of survival of 4 years suggested similar efficacy for each arm [23]. For patients in which the NSCLC tumor was medically inoperable, SBRT was a standard treatment [24,25]. Thoracic grade 3 or advanced AEs after 30 Gy in one fraction (arm 1) and 60 Gy in three fractions (arm 2) were compared using the Common Terminology Criteria for Adverse Events (CTCAE). It was established that 30 Gy delivered in one fraction corresponded to 60 Gy delivered in three fractions when observed in terms of life expectancy, PFS, toxicity, and OS. A single fraction SBRT was found to be more effective in terms of social functioning and dyspnea measures of QOL [26]. According to a secondary analysis of RTOG 0617, higher radiation doses were related to worse control of tumor and OS in stage III NSCLC. An independent cohort of patients treated at the University of Colorado School of Medicine was studied to determine the influence of the estimated dose of radiation on immune cells (EDRIC). After the definitive treatment of stage III NSCLC, higher radiation doses to the immune system were associated with the progression of tumors and death [27]. In the past, radiation therapy has been observed to produce out-of-field tumor regression (abscopal response), but it has recently gained significant importance with high-precision radiation delivery devices and has been used to treat various cancers, including NSCLC. Radiation therapy-induced abscopal effects in patients with advanced NSCLC were reviewed in a detailed study. When radiation therapy was used in combination with immunotherapy to treat advanced NSCLC or other types of cancers, the results indicated that radiation therapy could induce abscopal effects along with improved potential for boosting these effects. As a result of clinical trials investigating radiation therapy-induced abscopal effects, the use of radiation therapy for advanced NSCLC may be drastically changed, especially when combined with immunotherapy [28].

In peripherally located lung tumors, stereotactic MR-guided adaptive radiation therapy (SMART) has been found to be beneficial for delivering SABR. On an MR Linac or Cobalt-60 system, 23 patients (25 peripheral lung tumors) received SMART in 3–8 fractions. The on-table plan was adapted grounded on the anatomy of that day after each breath-hold MR scan. Under continuous MR guidance, breath-hold gated SABR was delivered using an in-room monitor, resulting in significantly smaller target volumes than if an ITV-based approach had been used. Despite ensuring ablative doses in all fractions with on-table adaptation, dosimetric benefits from daily online plan adaptation were modest in most peripheral lung cancer patients [29].

### 3.2. Chemoradiation Therapy

Chemoradiation therapy (CRT) was not appropriate for all stage III NSCLC patients. Sequential concurrent CRT had a high local failure rate, and therefore, intensification of treatment was justified. A multicenter feasibility study of intense modulated radiation therapy (IMRT) combining hyperfractionation, acceleration, and dose escalation was conducted. The study included patients with unresectable stage III NSCLC who had a performance status (PS) of 0 to 2 and were not eligible for concurrent chemotherapy. In 14 patients (37.2%), a maximum dose of 79.2 Gy was achieved. Esophagitis grade 3 was confirmed in two patients, but no pneumonitis grade 3 to 4 was reported. In addition to acute radiation pneumonitis, there were bronchopulmonary hemorrhages and acute lung infections of grade 5. In this study, the median survival time was 18.1 months (95% confidence interval [CI], 13.9–30.6), the two-year overall survival rate was 33.6% (95% CI, 17.9–50.1), and the PFS rate was 23.9% (95% CI, 11.3–39.1%) [30]. The rates of toxicity for curative and palliative radiotherapy were assessed and compared using a meta-analysis. Comparatively to individual trials, this provided more accurate quantitative assessments of toxicities. An analysis of randomized trials with >50 patients suffering from unresectable NSCLC who received palliative or curative conventional radiotherapy (RT) with or without chemotherapy was included in this systematic review. Among the data extracted were data on pneumonitis, esophagitis, pulmonary fibrosis, cardiac events, myelopathy, and neutropenia, as well as treatment-related deaths. However, the toxicity rate increased with the intensity of radiation therapy, and there was no significant difference between concurrent and sequential CRT when it came to esophagitis. Clinicians can use this information when making decisions about radiotherapy for NSCLC [31].

Radiation therapy (RT) is effective in treating many malignancies and relieving tumor-related symptoms. RT may, however, produce toxicity because surrounding tissues and organs are exposed to its biophysical effects [32]. Affected organs’ anatomy and physiology play a role in determining the manifestation of toxicity. The dose and volume of radiation applied to normal tissues usually have a direct relationship with the risk of toxicity, which has led to the establishment of guidelines and recommended dose limits for a majority of tissues. In addition to the characteristics of the patient at baseline as well as other treatments that are administered to them, side effects are multifactorial. These limitations of RT make it difficult to implement on a case-to-case basis.

## 4. Combination of Radiation and Chemotherapy for Lung Cancer Management

In certain conditions of lung cancer, only chemotherapy or only radiation was not sufficient to manage the disease. In such conditions, both chemotherapy as well as radiation were utilized for the tumor progression retardation. The use of definitive concurrent chemoradiotherapy (cCRT) should be considered for patients who have unresectable stage III NSCLC showing good status of performance. A meta-analysis of two large phase 3 randomized studies established the dominance of cCRT over sequential chemoradiotherapy (sCRT). Although cCRT offers greater efficacy, it is associated with more acute toxicity than the sequential treatment. There are currently a number of documented approaches to address this drawback. By using a multidisciplinary team (MDT) approach, the optimal treatment strategy can be determined at the point of diagnosis to minimize risks. Clinical oncologists can also find additional recommendations for defining target volumes and organs at risk in definitive cCRT (and adjuvant radiotherapy) by reviewing the guidelines of the Advisory Committee on Radiation Oncology Practice (ACROP). In addition, radiation oncologists could safely treat the larger tumors of the lung using high radiotherapy doses, resulting in greater accuracy, by utilizing modern advances in radiation therapy treatment planning and delivery. This resulted in reduced radiation dose to healthy tissues surrounding the lung tumor. As a result of these advances in cCRT, comprehensive strategies may be devised to allow the patients to benefit from potentially curative treatment modalities like immunotherapy and minimize risks associated with those treatments [33].

During radiation therapy for lung cancer, it is essential for patients to prevent pulmonary toxicity. Exercise training is not well established for patients with unresectable stage III lung cancer who are candidates for radical treatment. A home-based pulmonary rehabilitation (PR) program was evaluated to determine whether it was feasible to improve respiratory function, exercise capacity, and quality of life through the use of reliable tools. First, 20 patients (interventional group, IG) received PR concurrently with radiation therapy, while another 20 patients were identified as an observational group (OG). The 6 min walking test was performed at baseline (T0), followed by the modified Borg Scale (mBORG), the SF-36 questionnaire (SF-36), and the pulmonary function test (PFT) 8 weeks after the baseline (T2) and 4 weeks after the baseline (T1). After 4 weeks (T1), only the SF-36 questionnaire was administered. Due to the input from the OG, the mBORG scores trended downward; the IG scores, however, showed a slight improvement. There was a decrease in all items of the SF-36 score between T0 and T1 in the OG. There was an increase in the trend from T0 to T2 for all SF-36 items in the IG. There were no clinically significant differences in PFT between the baseline and T2 in either group. Thus, in assessing the effectiveness of PR programs, the 6MWT, mBORG, and SF-36 proved useful. Radio(chemo)therapy resulted in a significant increase in the capacity for functional exercise and a reduction in physiological impairment related to the quality of life [34]. Extensive-stage small cell lung cancer (ES-SCLC) patients have poor survival rates. The combination of cTRT with chemotherapy and upfront immunotherapy along with chemotherapy improved the outcome of patients incrementally but has not been evaluated in a clinical trial yet. After chemotherapy and immunotherapy, cTRT was used to characterize outcomes and toxicities. In two hospitals, researchers identified ES-SCLC patients treated with first-line chemotherapy, immunotherapy, and cTRT. The following outcomes were assessed for patients: PFS, OS, distant progression-free survival (DPFS), local progression-free survival (LPFS), and toxicity. It was observed that the first-line chemoimmunotherapy, which was followed by cTRT, appeared to be safe and produced comparable outcomes as found in published modern clinical trials. In order to determine whether cTRT is beneficial after chemoimmunotherapy, further studies are warranted [35].

SBRT, along with full-dose systemic chemotherapy, was studied in a phase 2 study (ClinicalTrials.gov NCT02568033) for unresectable stage 2 and stage 3 NSCLC. Toxicology and disease-free survival were the primary endpoints. SBRT was administered to all sites of gross disease. There were three fractions of 60 Gy given to peripheral lung tumors, five fractions of 50 Gy for central lung tumors, and five fractions of 40 to 50 Gy for hilar and mediastinal lymph nodes. A total of four cycles of chemotherapy was administered for nonsquamous histology, cisplatin and docetaxel for squamous histology, and cisplatin and paclitaxel for melanoma. In between cycles of chemotherapy, SBRT was given. SBRT was followed by chemotherapy after a seven-day break. Functional assessment of cancer therapy was used to measure the quality of life. SBRT, in combination with full-dose chemotherapy, appeared to be effective and safe for locally advanced NSCLC treatment [36]. The above studies could not conclude whether the combination of radiotherapy and chemotherapy resulted in lower toxicity. There are many limitations of non-specific radiation exposure to benign cells regarding mutation induction, DNA strand breaks, generation of reactive oxygen species, ionization, etc.

## 5. Other Treatment Strategies for Lung Cancer

Apart from chemotherapy and radiation therapy, there are other treatment modalities for the management of lung cancer. As a neuroendocrine tumor of the lung, SCLC is a potentially aggressive disease that has a metastatic tendency quite early in the course of the disease. The VA staging categorizes the disease as (a) limited stage (LS), which can be confined to one hemithorax and radiated in the same field or (b) extensive stage (ES), which is extended beyond one hemithorax. LS disease is currently treated with concurrent chemoradiation, and ES disease with chemotherapy alone. The current standard treatments will only cure a quarter of patients with LS disease, and most of the patients will eventually succumb to their disease. Despite SCLC’s resistance to conventional therapy and high recurrence rate, a complex genetic landscape provides the basis for effective targeted therapies. The potential roles for several different therapeutic strategies and targeted agents in SCLC have been investigated in recent years. Some of these agents have failed to show a survival advantage in this disease, including BCR-ABL TKIs, mTOR inhibitors, EGFR TKIs, and VEGF inhibitors. In addition, DNA repair inhibitors, antibody-drug conjugates (ADCs), immune therapy with vaccines, cellular development pathway inhibitors, immunomodulators, and immune checkpoint inhibitors are being tested. It is important to note that none of these agents have been approved to be used in SCLC, and most of them are undergoing phase I/II clinical trials, with immune checkpoint inhibitors as the most promising candidates [37].

### 5.1. Immune Checkpoint Inhibitors in Lung Cancer

In both second- and first-line settings, immunotherapy has demonstrated superior efficacy over chemotherapy alone in treating advanced NSCLC. However, only 20% of patients respond to checkpoint blockade, so novel insights into molecular mechanisms and regimens are needed to improve immunotherapy’s effectiveness. The immune checkpoint inhibitors, combined with chemotherapy, seem to be an effective strategy to prevent tumor cells from evading the immune system through cancer immunoediting. The strategies are: (1) enhance the immune response against tumor cells (immunogenic cell-death), and (2) reduce the immunosuppressive environment around tumors. In combination with chemotherapy, the immune checkpoint inhibitors atezolizumab and pembrolizumab are FDA-approved and recommended already as the first-line treatment for advanced NSCLC. Moreover, as an initial therapeutic approach for metastatic NSCLC, many other chemo-immunotherapeutic regimens have also been evaluated. At the same time, numerous preclinical studies have examined the molecular mechanisms of chemotherapeutic agents used conventionally (antimetabolites, anthracyclines, antimitotic agents, and platinum salts), unraveling effects of drugs and doses/schedules on the immune system that can be exploited for synergistic clinical outcomes [38,39]. The first-line treatment for advanced NSCLC has been changed by immune checkpoint inhibitors (ICIs), which target the PD-1/PD-L1 axis. For patients whose PD-L1 expression is less than 50%, pembrolizumab (a PD-1 inhibitor) is recommended as a monotherapy or combined with chemotherapy. Bevacizumab (an anti-angiogenic antibody) and atezolizumab (PD-L1 inhibitor) can also be used in combination with chemotherapy for first-line treatment of NSCLC regardless of PD-L1 expression. People with high tumor mutational burden (TMB) may also benefit from PD-1/PD-L1 inhibitors in combination with anti-CTLA-4 antibodies for advanced NSCLC compared to platinum-based chemotherapy. For all patients with PD-L1 expression of 1% or more, the FDA has approved the combination of ipilimumab (an anti-CTLA4) and nivolumab (a PD-1 inhibitor). Compared to chemotherapy, immunotherapies alone or in combination with chemotherapy prolong life in people with advanced NSCLC. Chemotherapy may have a higher frequency of side effects than immunotherapy alone. In spite of the widespread use of these antibodies in clinical practice, a few questions remain regarding the best strategy for treatment, the effectiveness of immunotherapy, and the role of different biomarkers in treatment selection, depending on the patient’s clinical characteristics [40,41].

A network meta-analysis (NMA) comparing the efficacy of immune checkpoint inhibitors (ICIs) with or without chemotherapy in metastatic NSCLC patients was conducted based on 12 phase-III studies involving 9236 patients. Combined direct and indirect evidence was analyzed in the NMA, including the results of randomized studies with chemotherapy as the common comparator. Using a frequentist NMA, the hazard ratio (HR) of PFS was estimated. Compared with other treatments studied, chemotherapy combined with pembrolizumab and atezolizumab produced the highest PFS within the overall cohort. Squamous and non-squamous patients both benefited from this superior PFS. Based on non-squamous histology, the pembrolizumab/chemotherapy combination and atezolizumab/bevacizumab/chemotherapy (ABC) provided the best overall survival results in the overall cohort. Again, chemotherapy in combination with atezolizumab or pembrolizumab exhibited significant benefits of PFS, followed by monotherapy using pembrolizumab in patients with high PD-L1 levels. For advanced NSCLC patients, chemotherapy combined with ICIs enhanced treatment efficacy. Compared to chemotherapy alone or any other ICI combination or monotherapy, chemotherapy combined with pembrolizumab or atezolizumab consistently showed higher efficacy in non-squamous cancers [42]. Patients with advanced malignant neoplasms, such as metastatic non-small cell lung cancer (mNSCLC), may benefit from new intermediate endpoints to detect early activity and prioritize new therapies. A study involving more than 150 patients, whose intention-to-treat population was identified, was submitted to the US Food and Drug Administration to examine the milestone rate, an intermediate endpoint for immunotherapy trials. Trial-level milestone ratios were estimated for the overall response rates (ORRs) within 6 months, 9-month PFS rates (PFSs), and 9-month OS rates (OSs). They evaluated the association between milestone ratios and hazard ratios (HRs) using a weighted linear regression model. The Kaplan–Meier survival estimates were compared between the experimental and control arms of trials testing the targeted therapy, immunotherapy, and other therapies. Compared to PFS or 6-month ORR milestones, OS milestones at 12 and 9 months had a moderate association with OS HR. In future trials, however, where immunotherapy may increasingly be the control, new biomarker-enrichment strategies will be deployed, patients with lengthier survival are likely to enroll, and OS HR may not be the optimal time. As a complementary tool or as a secondary outcome in exploratory studies, milestone rates can provide useful information about trial results [43].

### 5.2. Gene Therapy

In order to treat lung cancer, two novel approaches have been proposed, including gene therapy and immunotherapy. Preclinical data suggest that both treatments may have potential clinical applications. It has been discovered that specific genes are critical to the development of carcinogenesis and that these genes or their products can be targeted for treatment as part of gene therapy programs. As a possible gene therapy strategy, it has been suggested that the adenoviral gene transfer technology (Ad-p53) could be used for the replacement of nonfunctional tumor suppressor genes, like the deleted or mutated p53 genes, with wild-type p53 genes in phase I and phase II lung cancer trials. Direct intratumoral injection and bronchoalveolar lavage have been used in order to achieve transduction of the tumors. By combining Ad-p53 with radiation or perhaps even chemoradiation, these studies have demonstrated a budding role for the radiosensitization of formerly radiation-resistant local tumors [44]. There has been a gradual delineation of the genetic etiology of cancer in the last three decades, but it still has not been completely described. Having a better understanding of the molecular events that take place during the multistep process in bronchogenic carcinogenesis could help us to overcome these challenges with greater ease. There has been a great deal of progress made in these three decades when it comes to developing methods for transferring functional genes into mammalian cells. A gene therapy can, for example, prevent the activation of tumor-promoting oncogenes or replace inactivated genes that promote apoptosis or tumor suppression with other tumor-suppressing or apoptosis-promoting genes. It has been discussed by researchers how these molecular changes associated with bronchogenic carcinomas may have therapeutic implications. It has been found that Ras, Erb B-2 (HER-2/neu), Erb B-1 (EGFR), fur, myc, fes, raf, sis, Bcl-2, Bcl-1, and IGF-1 genes may be altered in NSCLC. As far as the therapeutic gene to be transferred is concerned, it falls into one of six categories: RNAi, antisense, or ribozyme sequences against oncogene transcripts; cytokine genes; replacement of tumor suppressor gene; cell surface antigens; suicide genes; as well as multidrug-resistant genes. It is possible to correct the abnormal malignant phenotype by inhibiting the oncogene or by replacing the tumor suppressor gene. Transduced tumor cells would be able to produce toxic metabolites by enzymatically converting otherwise non-toxic substances. It is also possible that the transferred gene will allow cytotoxic drugs to penetrate drug-resistant tumor cells. In order to improve tumor/immune cell interaction and to stimulate the immune response, it would be beneficial if the genes for tumor-specific antigens, MHC molecules, adhesion molecules, co-stimulatory molecules, or cytokine molecules were delivered [45,46].

**Figure 3:**
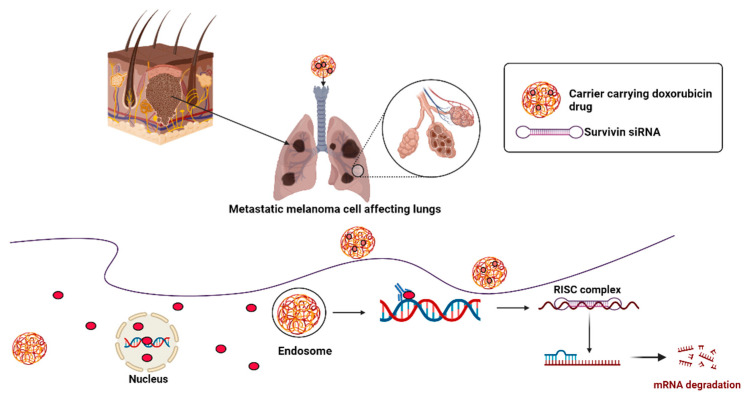
The delivery of doxorubicin and Survivin siRNA in lung cancer cells using a nanopolymer- based drug delivery system. The drug and siRNA are taken up by the cells via endocytosis. The siRNA makes the RISC complex, thereby degrading the mRNA. The image was recreated from ref. [50].

Apart from immunotherapy and gene therapy, there are a few additional therapeutic approaches developed by researchers. There has been an increase in interest in cancer stem cells (CSCs) in recent years. Essentially, CSCs can self-renew as well as differentiate into different types of cells in order to generate new tumors. Numerous studies report that CSCs mediate tumor recurrence and are resistant to many conventional therapies. A number of markers, such as CD133, CD44, ABCG2, and ALDH1A1, can be used in order to detect CSCs in lung cancer, as well as other characteristics of CSCs, including spheroid formation and colony formation. A potential approach for inhibiting tumor progression would be to target these surface proteins using blocking antibodies and to inhibit ABC transporters and the aldehyde dehydrogenase (ALDH) enzymes utilizing small molecules. There are three signaling cascades that govern the fate of cells during development, the Hh, Notch, and Wnt cascades, and these pathways are involved in the formation of CSCs in a variety of solid tumors. It has also been found that therapeutic approaches can target these signaling pathways in order to inhibit the progression of tumors [47]. A pilot study was conducted to evaluate the efficacy of electrochemical treatment (ECT) as the therapy for 386 patients with NSCLC. In this study, two different ECT methods were employed: firstly, platinum electrodes were introduced transcutaneously inside the tumor under the guidance of X-ray or CT in cases of peripherally located lung cancers. The electrodes were inserted intraoperatively, directly into the tumor for the treatment of central-type lung cancers or for those that could not be operated on during thoracotomy. There were 6–8 V of voltage, 40–100 mA of current, and 100 coulombs of electric charge per cm of tumor diameter. Since the effective area around each electrode was approximately 3 cm in diameter, the number of electrodes was determined by the size of the cancer mass. The clinical results showed that ECT was a simple, safe, and effective therapy with minimal trauma. When lung cancer was conventionally inoperable, unresponsive to chemotherapy or radiotherapy, or unresectable following thoracotomy, ECT offered an alternative treatment option. Further research into ECT is warranted based on the long-term survival rate of treated patients [48]. Despite their potential as cancer therapeutics, microRNAs and siRNAs have been challenging to deliver to most solid tumors. A study showed that a new lung-targeting nanoparticle could deliver miRNA mimics as well as siRNAs to the lung adenocarcinoma cells and tumors in a mouse model of lung cancer whose Kirsten rat sarcoma viral oncogene homolog (Kras) was activated and whose p53 function was lost using genetic engineering. As a result of the therapeutic delivery of miR-34a, a tumor suppressor miRNA that is regulated by p53, miR-34a levels were restored in lung tumors, miR-34a target genes were specifically down-regulated, and tumor growth was slowed. Kras gene expression and MAPK signaling were reduced through siRNA delivery, apoptosis was increased, and tumor growth was inhibited by the delivery of siRNAs targeting Kras. Tumor regression was improved by combining miR-34a with siRNA targeting Kras, compared to either small RNA alone. Further, in this model, chemotherapy plus nanoparticle-based small RNA delivery prolonged survival, compared to chemotherapy alone. As a result of these findings, researchers provided preclinical evidence that small RNA therapies could be used in cancer patients and allowed RNA combination therapy in an autochthonous lung cancer model [49]. Doxorubicin (DOX) and Survivin siRNA have been delivered through a pH-sensitive delivery system to treat metastatic lung cancer. Polyethylenimine-BMPH-DOX (PMD) conjugates were made via a pH-sensitive hydrazine bond (3-aleimidopropionic acid hydrazide, BMPH). B16F10 cells were successfully transfected with DOX and Survivin siRNA and their cytotoxicity was enhanced. There was a preferential accumulation of DOX and siRNA in the lungs of B16F10 tumor-bearing mice after local delivery of PMD/siRNA nanoparticles by pulmonary delivery. In the tumor tissues of the lungs, a considerable amount of DOX and siRNA were observed, whereas limited amounts of DOX and siRNA were observed in the normal tissues of the lungs. Overall, these findings provide a promising strategy for the treatment of metastatic lung cancer by using pulmonary administration as a local delivery method [50]. A schematic representation of the possible killing of the cancer cells by Survivin siRNA is shown in Figure 3.

There has been limited success with radiotherapy alone in treating the lung cancer patient population. In a previous study, radiofrequency ablation (RFA) was applied to lung tumors as an image-guided, thermally mediated ablative technique. There has never been a combination therapy that combined both of these treatments before. According to the researchers, a combination of CT-guided radiofrequency ablation along with conventional radiotherapy was used in 24 medically inoperable patients, with a minimum follow-up of 2 years for the survivors. During the course of the treatment, 24 consecutive patients with biopsy-proven, stage I NSCLC who were medically inoperable were treated with CT-guided RFA, followed by 66 Gy of radiotherapy in a dose-dependent manner. In this study, RFA was performed using a single or cluster of cool-tip F electrodes, and fluorodeoxyglucose, a radioactive tracer, was used to stage 21 patients before therapy was administered. For patients with medically inoperable stage I NSCLC, RFA followed by conventional radiotherapy may be feasible. Despite the addition of RFA, few procedural complications, and low levels of major toxicities, radiotherapy alone appeared ineffective in controlling local disease and achieving a better survival rate [51].

The above therapeutic strategies need further improvement to alleviate the suffering of lung cancer patients during these treatment interventions and improve their efficacy. Nanotechnology has become popular in the last few decades for improving the effectiveness of chemotherapeutic drug delivery as well as improving radiation therapy efficacy.

## 6. Lung Cancer Chemotherapy Using Nano-Enabled Drug Delivery System

**Figure 4:**
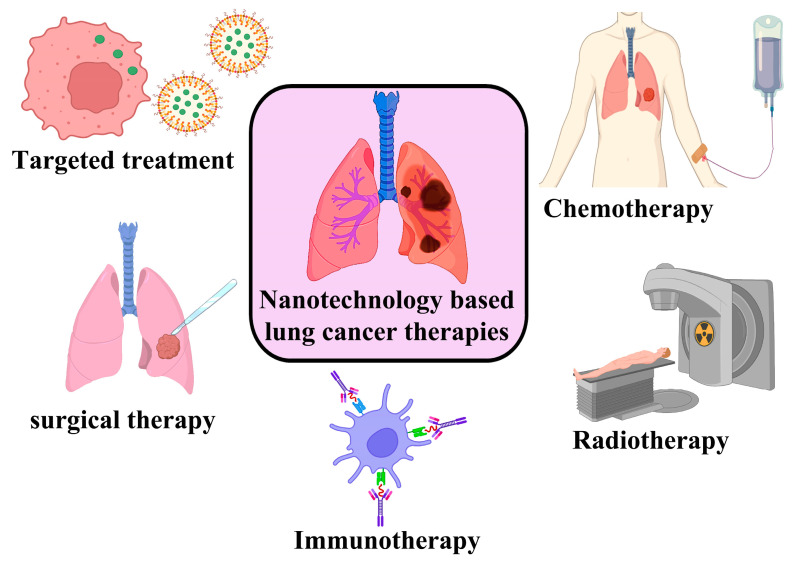
The applications of nanotechnology-based therapeutic approaches in lung cancer including targeted drug delivery, improving chemotherapy and surgical resection, radiotherapy and immunotherapy.

Despite its effectiveness in treating both types of lung cancer, chemotherapy still has severe limitations. The majority of drugs used in chemotherapy damage not only actively dividing cells but also damage the healthy cells in the digestive tract, constantly dividing cells in hair follicles, and bone marrow. The reticuloendothelial system is also adversely affected by anticancer drugs [52]. For the diagnosis and treatment of lung cancer, a variety of nanoparticle systems have been developed, including organic, inorganic, metallic, and polymeric nanoparticles [53]. Gholami et al. discussed the recent research and also various ongoing studies in the clinical application of dendrimers, liposomes, and polymeric micelle nanoparticles for the management of lung cancer [54]. Its interaction with the surface of the respiratory system plays a significant role in determining the toxicity of the nanoparticle. The potential toxicity of nanoparticles to the pulmonary surfactant, alveolar epithelium, and immune system should be considered in developing new nanoformulations for lung cancer treatment, and this has been discussed by researchers [55]. The different applications of nanoparticles in lung cancer therapy are shown in Figure 4.

Magnetic resonance imaging (MRI) guided nanoparticle therapy has also been reported in the literature [56,57,58,59]. Since nanoparticles have several advantages over chemotherapeutics, they have been employed for lung cancer therapy to overcome the aforementioned chemotherapeutic-associated problems. As a result of enhanced permeability and retention (EPR) of nanoparticles, they preferentially accumulate drug-loaded nanoparticles within tumor cells, and they can encapsulate and deliver drugs that are not easily dissolved [60,61]. In spite of the advancement of nanoparticle-based drug delivery systems, chemotherapy still faces challenges. Multidrug resistance (MDR) is most often caused by drug-resistant genes overexpressed by cancer cells that resist anticancer drugs and pump the drugs outside the cells. Due to this, higher doses of drugs are required to kill cancer cells, increasing the risk of adverse effects. SiRNAs are double-stranded short RNAs of 21–24 nucleotides that guide the endonucleolytic cleavage of mRNAs according to their sequence. As potential new cancer drugs, siRNAs have a number of important advantages [62]. Since siRNAs theoretically can be designed to target any known gene, they are potentially useful in treating a wide range of cancers caused by one or a few genes. It is possible to downregulate oncogenes with a lower chance of off-target effects because siRNAs have a high sequence specificity, enabling them to distinguish between even single nucleotide mismatches [63]. SiRNAs have several drawbacks: their size (13 kDa) and net negative charge make them difficult to cross the cell membrane; they are susceptible to enzyme digestion (RNase) and are quickly excreted through kidney filtration. A number of strategies for delivering siRNAs have been developed to overcome these limitations, especially nanotechnology, which has provided several advantages in the field of RNAi therapeutics, including siRNA protection, biocompatibility, ease of scaling and modification, improved quality control, and storage stability [64]. In order to improve the stability, bioavailability, and retention of the anti-cancer drugs and siRNAs in the targeted lung regions, nanoparticle-based drug delivery systems like the polymeric, lipid, micellar, inorganic, and dendrimer nanoparticles are being developed and evaluated as potential delivery systems [65,66,67].

Liposomes are used for the delivery of siRNA and contain cationic lipids such as N-[1-(2,3-dioleoyloxy)propyl]-N,N,N-trimethylammonium methyl sulfate (DOTAP) and N-[1-(2, 3-dioleyloxy)propyl]-N,N,N-trimethylammonium chloride (DOTMA). Positively charged head groups, hydrophobic tails, and linker groups make up cationic lipids. It has been reported that TF pathway inhibitors suppress lung metastasis, which led Amarzguioui et al. to deliver tissue factor siRNA using Lipofectamine 2000 in pulmonary tumor mice. A dramatic reduction in the incidence of pulmonary tumors was observed in C57BL/6 mice after intravenous (iv.) injection of cells that were transfected with TF siRNA/Lipofectamine 2000 complexes, suggesting that TF siRNA could be used as a clinical strategy to prevent lung tumor metastasis [68]. To overcome the limitation of deprived siRNA cellular uptake for clinical use, Zhang et al. delivered HDM2 siRNA using polyarginine (R8) modified liposomes. Polyarginine is an example of a cell-penetrating peptide that can penetrate cell membranes. In addition to showing considerable stability against degradation in blood serum, the R8-modified liposomes/HDM2 siRNA complexes also significantly reduced lung tumor cell proliferation due to the assimilation of R8 in the liposomes, but they did not deliver active siRNA [69]. Another study explored the synergistic effects of 7-O-geranyl quercetin (GQ) in combination with IGF-1R siRNA (siIGF-1R) transported inside a liposome toward human NSCLC. As a first step, GQ was loaded inside cationic liposome CDO14 in order to form CDO14-GQ before being united with siIGF-1R to form a liposome for co-delivery, named CDO14-GQ-siIGF-1R. In a Western blot assay, it was shown that CDO14-GQ-siIGF-1R caused a significant decrease in the levels of expression of siIGF-1R in cells and tumor tissues, as well as a stronger effect on the expression of Bcl-2 and Bax, the apoptosis-related proteins, in comparison to CDO14-GQ or CDO14-siIGF-1R. According to these findings, co-delivery of GQ and siIGF-1R via liposomes enhanced the tumor regression effect of either of these drugs when delivered together. Based on the results of this study, it is evident that combining siRNAs with chemotherapeutic drugs is an effective strategy for treating NSCLC [70]. In a recent study, it was found that a new peptide ligand, CP7, was capable of binding to FGFR1 via reverse molecular docking and could unite with VEGFR3 to target A549 cells through its interaction with each receptor. As a result of the modification of CP7 on the liposome surface, a targeted and benign nano vehicle was constructed for the delivery of Mcl-1 siRNA, which is a therapeutic gene that was incorporated into the liposome. There was significant apoptosis of tumor cells in vitro as a result of siRNA-loaded liposome-PEG-CP7 uptake because of specific binding amid CP7 and A549 cells. This was due to the Mcl-1 gene silencing, which was connected with angiogenesis and apoptosis, through siRNA loading. As a result of the gene delivery system in vivo, tumor-bearing mice displayed significantly better antitumor activity than those without tumors. Based on all of these findings, siRNA-loaded liposomes coated with PEG-CP7 with good bioavailability and minimal side effects might serve as a promising system for gene delivery possessing good bioavailability and low side effects [71]. An improved delivery system (L-PTX-PSur) has been developed to efficiently co-deliver survivin siRNA (Sur) and paclitaxel (PTX) to circumvent the dose-limiting toxicity and achieve enhanced therapy by the synergistic effect of PTX and Sur. In this study, a carbamate linked with cationic lipid was engineered to make PTX-loaded liposomes that encapsulated the siRNA, and protamine was used to condense the siRNA into a compacted "core". L-PTX-PSur suppressed survivin protein expression in NCI-H460 cells by a noticeable amount in Western blot analysis. PTX’s effectiveness at low doses could be enhanced by down-regulating survivin protein, which could reduce cancer cells’ apoptotic threshold. A low dose of PTX combined with L-PTX-PSur could cause PTX and Sur to work synergistically to inhibit cancer cell growth. The results of this study provided a promising strategy for treating lung cancer [72].

Despite the fact that about 16 liposomal drugs are available on the market as of now, only a few formulations are FDA-approved for the treatment of NSCLC [73,74]. It was reported by Mukherjee et al. that a series of guanidinylated cationic amphiphiles were designed and synthesized in order to inhibit the growth of B16F10 solid tumors. It was established that the systemic administration of synthetic CDC20 siRNA entrapped in liposomes of a guanidinylated cationic amphiphile with stearyl tails inhibited the growth of the tumor. In a syngeneic C57BL/6J mouse tumor model, administration of the same liposomal formulation intravenously inhibited the growth of B16F10 melanoma cells on the lungs (metastases) [75]. Researchers developed a multifunctional targeting liposome to treat NSCLC with better in vivo effects. On the liposome surface, Octreotide (OCT), a synthetic analogue of somatostatin, was used to bind somatostatin receptors overexpressed in various tumors. As part of the study, the co-encapsulation of two drugs was executed within the liposomes: Honokiol into the lipid bilayer in order to reduce the metastasis of tumor and inhibit the formation of vascular mimicry channels, and epirubicin inside the aqueous core as a drug for inhibiting tumor. The results of mechanistic studies have shown that the liposomes have the ability to inhibit PI3K, MMP-2, MMP-9, VE-Cadherin, and FAK, as well as to activate caspase-3 [76].

Deoxyribonucleic acid (DNA) and ribonucleic acid (RNA) sequences are delivered to cancer cells through nanoparticles composed of proteins, polysaccharides, artificial polymers, and lipids. In addition, through the modification of the surface of nanoparticles or the conjugation of biomolecules with the surface of nanoparticles, the effectiveness of cancer targeting has been enhanced [77]. The most common type of nanostructures used for drug delivery are polymers that can be natural or synthetic. There are a number of polymer systems that are used in therapeutics for lung cancer, such as poly(lactide-co-glycolide) (PLGA), poly(ε-caprolactone) (PCL), gelatin, polylactic acid (PLA), alginic acid, and chitosan [78]. PLGA was used to deliver Paclitaxel in HeLa cells and NMRI mice, 9-Nitro-camptothecin in PBS, and pDrive-sh AnxA2 plasmid DNA in mice [79,80,81]. PEI and PEG-PEI copolymer were used to carry pCMV Luc DNA and deliver it in mice through intravenous injection, and in A549 and Calu-3 cells, and also in preclinical mice, respectively [82,83]. PEI was also used as a carrier to deliver p53 plasmid using intravenous injection as well as aerosol inhalation in mice and B16-F10 tumor-bearing mice, respectively, to reduce lung cancer [84,85]. PEG-PLGA was used to deliver NF-κB decoy in rat models and the explanted lungs from PAH patients [86]. Poly-L-lysine (PLL), improved with N-terminal cysteine-polyethylene glycol, was used to infuse *Escherichia coli* genomic DNA into mice via the intranasal route [87]. PEG-substituted PLL delivered Firefly luciferase intranasally into C57BL/6 mice for cancer imaging [88]. PEGylated gelatin nanoparticle administered pCMV β-gal intravenously and intratumorally into LLC-bearing female C57BL/6 J mice for therapy [89]. The polyplexes of chitosan oligomer were used for encapsulating FITC-labeled pCMV-Luc and delivered to HEK 293 cells for theranostics [90]. Solid lipid nanoparticles were used as cargo to deliver calcitonin, cyclosporine A, and somatostatin for administration via parenteral routes or through nasal, pulmonary, and oral routes in rats [91]. Branched polyester was utilized to deliver 5(6)-Carboxyfluorescein into a rabbit lung model [92]. Poloxamer-188 and glycerol carried T cell-specific surface antigen to Human bronchial Calu-3 cell line [93]. Researchers reported on the effectiveness of nanoparticles made from polycaprolactone (PCEC)/polycaprolactone/poly (ethylene glycol) loaded with paclitaxel (PTX) for the treatment of lung cancer. By highlighting the crucial role of circadian rhythms in cancer propagation, the authors attempted to map the most appropriate time of day to administer nano-carriers loaded with the drug. It was found that 15HALO is the most effective chemotherapy for tumor growth inhibition in vivo [94]. Wang et al. recently used mesenchymal stem cells to deliver nanoparticles carrying docetaxel (DTX) in order to overcome the low targeting capacity of the nanoparticles. Drug loading by MSCs proved superior to that by fibroblasts. Animal and cellular experiments proved that nanoparticles were transported intercellularly from MSCs to cancer cells. In vivo, they inhibited primary tumor growth as well [95].

In recent years, researchers have been focusing on bio-nanoparticles with high biocompatibility, better stability, and biodegradability, including solid lipid nanoparticles, protein nanoparticles, aptamers, viral nanoparticles, and apoferritin, wherein biomimicking components are incorporated into the therapeutic nanoparticles. Nanoparticles of these types have been successfully engineered and utilized for the theranostics of cancer in the past [96,97,98,99]. Gold, carbon dots, silver, silica, rare-earth oxides, iron oxides, and nanodiamonds are among the various inorganic nanoparticles that have been extensively studied for their potential as cancer theranostics. The size, shape, surface charge, concentration, and time of exposure of these nanoparticles all significantly affected cytotoxicity in vitro and in vivo on different lung cells. By controlling these physicochemical parameters accurately, lung cancer theranostics can be made meaningful [77,99,100,101]. Other types of nanostructure-based drug delivery systems have been previously reviewed in detail by other researchers [99,102].

Two of the most prominent Doxorubicin liposomes, Doxil and Myocet, received FDA approval in 1995 and 1999, respectively, following several other liposomes in the same category. Paclitaxel–Albumin-stabilized Nanoparticle Formulation (Abraxane^®^) is a nanoformulated chemotherapeutic drug that has been approved by the FDA for NSCLC treatment [8]. There are a few nanoformulations of chemotherapeutic drugs for lung cancer that are undergoing clinical trials. Doxorubicin Hydrochloride (Adryamycin^®^, Rubex^®^), entrapped in pegylated liposome, is for IIIB-IV lung cancer and is in Phase II clinical trials; entrapped in aerosolized liposome, it is for IIIB lung cancer and is in phase I clinical trials; and entrapped in only liposome, it is for IIIB lung cancer and is undergoing phase IV clinical trials. Paclitaxel was encapsulated in polymeric micelle (Genexol-PM^®^) and administered to stage IV lung cancer patients in a phase II trial. Camptothecin was entrapped in aerosolized liposome for IIIB-IV lung cancer and is undergoing preclinical studies. Lurtotecan was encapsulated in liposomes for stage IIIB lung cancer and is undergoing a phase I clinical trial [8]. Table 1 shows the different nanoformulated chemotherapeutic drugs used/under clinical trials for the treatment of lung cancer [103].

## 7. Lung Cancer Radiation Therapy in Combination with Nanoparticles

**Figure 5:**
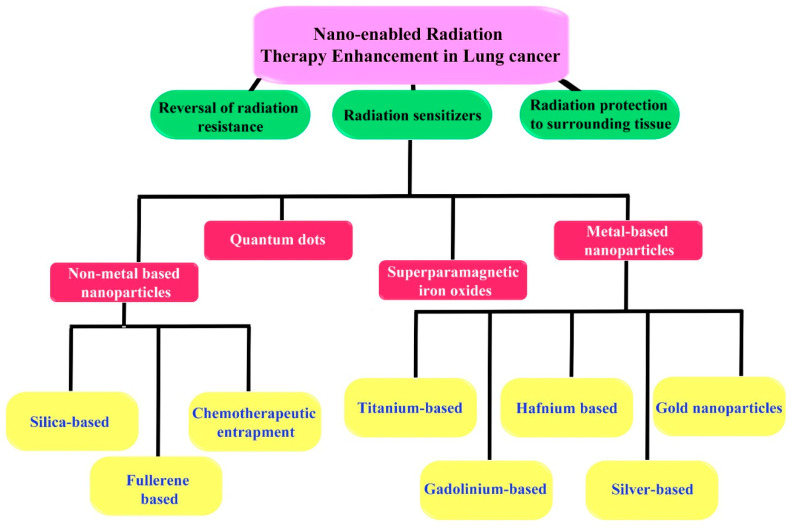
Enhancement of lung cancer radiation therapy using nanoparticles.

Ionizing radiation therapy has made great advances, including improved focusing and appropriate regulation of radiation dose, but some chief issues remain unaddressed. It is still a balancing act between the therapeutic advantages and physiological disadvantages of the therapeutic system due to radiation resistance and the intrinsic errors of the therapeutic protocol. In order to enhance efficacy while reducing toxicity, various approaches have been implemented. They include three major approaches: (1) enhancing tumor tissue radiosensitization; (2) reversal of tumor tissue radiation resistance; and (3) enhancing radioresistance in the tissues that are healthy. The nanoparticles-based strategies to improve the efficacy of radiation therapy are summarized in Figure 5 [104].

It is possible for X-rays to have multiple outcomes when they hit metal. There are several types of emissions that may occur during cancer radiotherapy, including scattered Auger electrons, Compton electrons, X-rays/photons, photoelectrons, and fluorescence photons. The incoming radiation ejects electrons from their orbitals with kinetic energy, which is equivalent to the wave energy minus the electron binding energy. Radiation from the electron determines its range within a tissue based on its kinetic energy. The photoelectric effect is determined by (Z/E)^3^, where E represents the photon’s energy and Z represents the molecule’s atomic number. Gold, because of its high Z (79) and inert property, is ideal for photosensitization [104].

Metal nanoparticles have been used for improving the radiosensitization of lung tumors, with gold nanoparticles (GNPs) being one of the major metal nanoparticles. As a model of NSCLC, Lewis lung carcinoma (LLC) was tested in vitro with the use of comet and clonogenic assays to investigate the potential radio-sensitization effects of two GNPs of different sizes (3.9 and 37.4 nm). In comparison to radiation and particles alone, both particle sizes demonstrated increased DNA damage following 2 Gy X-ray irradiation. In turn, this radio-sensitization led to a reduction in clonogenicity and cell survival. In vitro, both sizes of GNP induced DNA damage in LLC cells, but the 37.4 nm particles caused greater damage [105]. It was shown that thio-glucose-functionalized gold nanoparticles (Glu-GNPs), having a size of 13 nm, in combination with megavoltage (MV) X-rays, significantly inhibited the growth of human A549 lung cancer cells. By increasing the G2/M ratio and inducing more apoptosis, Glu-GNPs enhanced radiation effects. Additionally, glu-GNPs deregulated Bcl-2 when combined with radiation, whereas Bax and caspase 3 activity were increased. As a novel radiosensitizer, Glu-GNPs, in combination with radiation, could increase A549 cell cytotoxicity by arresting G2/M phases, as well as increasing apoptosis—presumably through regulation of the expression of the Bcl-2 family of proteins and mitochondrial apoptosis [106]. The effect of AuNPs loaded with small interfering RNAs (siRNAs)-SP1 on the radiosensitizing effect and mechanism of AuNPs-si-SP1 on lung cancer has been studied. By gel electrophoresis, AuNP adsorption to siRNA-SP1 was determined, and laser confocal microscopy was used to observe AuNPs-si-SP1 uptake. Western blot analysis and RT-qPCR were used to validate the silencing efficacy of AuNPs-si-SP1. The viability of cells was assessed with CCK-8 assays, radiosensitization with colony formation assessment, apoptosis and cell cycle analysis using flow cytometry, and DNA double-strand breaks using immunofluorescence in the absence or presence of AuNPs-si-SP1 or granzyme B (GZMB). Bioinformatics analysis predicted the SP1 downstream mechanism, and Western blot analysis verified it. For in vivo verification of AuNPs-si-SP1 and GZMB radiosensitization, subcutaneous tumorigenesis was performed on nude mice. By absorbing SP1 siRNA and internalizing it, AuNPs-si-SP1 reduced SP1 protein expression in A549 cells. Increased radiosensitivity was associated with AuNPs-si-SP1 and GZMB overexpression. Through the inhibition of SP1 to upregulate GZMB, AuNPs-si-SP1 inhibited solid tumor growth in nude mice to achieve radiosensitization. As a result of inhibiting SP1 for the upregulation of GZMB, lung cancer radiosensitivity may be elevated by AuNPs-si-SP1 [107]. Other metal and metal oxide nanoparticles used for improving radiosensitization include gadolinium, titanium dioxide, quantum dots of CaF, ZnS LaF, or ZnO, superparamagnetic iron oxide nanoparticles, silver, and hafnium oxide (HfO_2_). The non- metallic nanoparticles include polymeric nanoparticles, silica, fullerene, etc., that could improve radiosensitivity [104]. It has also been discussed how nanoparticle-based radiosensitizers and nanoparticles (NBRs/NPs) can be delivered intratumorally for the enhancement of radiosensitivity [108].

E-DSNPs (dual-stimuli nanoparticles) were designed by researchers and are composed of two parts: (1) core: a chemo-drug encapsulated in Cisplatin (a dual-stimuli nanoparticle), and (2) shell: a radiation sensitizer encapsulated in NU7441 (responsive to irradiation) as stimuli. As the target moiety for lung cancer cells, there was high expression of ephrin transmembrane receptor A2 (EphA2). As a result, the effectiveness of these nanoparticles against lung cancer cell lines was assessed. In comparison with healthy lung epithelial cells, E-DSNPs were highly taken up by lung cancer cells. In vitro, the cancer cell survival fraction was reduced by about 0.019 and 0.19, respectively, compared to free drugs of equivalent concentration when both drugs were released through these nanoparticles in response to respective stimuli. These engineered nanoparticles could potentially be used for targeted cancer therapy, thereby overcoming conventional clinical treatments’ side effects [109]. The different nanoparticles used for improving radiation therapy in different in vitro models of lung cancer are summarized in Table 2.

Apart from in vitro studies, there are many reports on in vivo models in which radiosensitization was enhanced due to the presence of nanoparticles. Menon et al. explored both in vitro as well as in vivo lung cancer models to determine the chemo-radiosensitization improvement. As mentioned in Table 2, in order to enhance localized chemo-radiotherapy to effectively treat lung cancer, Menon et al. developed a multifunctional dual drug-loaded nanoparticle that targets folate receptors and contains poly(N isopropylacrylamide)-carboxymethyl chitosan shells and polylactic-coglycolic acid (PLGA) cores. It was shown that these nanoparticles could be encapsulated with superparamagnetic iron oxide nanoparticles (SPIO) in order to visualize them using MRI in vivo in mice with H460 tumors. These particles demonstrate low toxicity in vivo and are suitable for use in chemoradiotherapy [114]. Radiotherapy (RT) faces two major challenges: insufficient radiation deposition in tumors and hypoxia-induced radioresistance. Using porous platinum nanoparticles, researchers proposed solving these two problems simultaneously with a single agent. By effectively displacing X-ray radiation energy into cancer cells (NCI-H460 cells), porous platinum nanoparticles could enhance the ROS stress, radiation-induced DNA damage, and arrest of cell cycle significantly due to the advantages of a combination of high-Z element and capacity to generate oxygen. Furthermore, platinum nanoparticles improved the oxygenation in the tumor by converting endogenous H_2_O_2_ to O_2_, increasing the RT without causing in vivo toxicity. Male athymic nude mice (Balb/c-nu, 5 weeks old) were implanted with lung cancer xenografts bearing NCIH460 cells subcutaneously inside their right hind flanks for anti-tumor activity assessment. Based on the oxygen generation properties of porous multi-Z metal nanoparticles, this study presented a novel nanomedicine strategy for synergistic enhancement of RT [118]. Researchers used NSCLC as a model disease to evaluate Genexol-PM, one of the lone clinically approved NP chemotherapeutics possessing a controlled drug release profile, as a radiosensitizer in preclinical studies for facilitating the clinical translation of NP chemotherapeutics to be used in chemoradiation therapy. NSCLC cell lines and mouse xenograft models were used to evaluate Genexol-PM’s efficacy as a radiosensitizer. After Genexol-PM administration, paclitaxel doses to normal lungs and liver were quantified and compared with those after Taxol administration. H460 and A549 cells were used to evaluate Genexol-PM as a radiosensitizer, and it was shown to be highly effective compared to Taxol, its smaller molecule counterpart, at half maximal inhibitory concentrations. Genexol-PM was shown to be more effective than Taxol as a radiosensitizer in mice bearing H460 tumors in an in vivo study. In addition, Genexol-PM reduced the exposure of paclitaxel to normal lung tissue at 6 h after administration compared to Taxol [119]. A combination of radiation and PD1 blockade suggested significant therapeutic benefits among numerous types of tumors; however, in many cases, anti-PD1 resistance prohibited these beneficial effects. The researchers combined radio-enhancing nanoparticles (NBTXR3) with localized radiation to achieve immunotherapy effects in a mouse model of lung cancer resistant to anti-PD1. There was significant growth delay in both irradiated and unirradiated tumors, when NBTXR3 was combined with localized radiation and systemic anti-PD1 in both the 344SQP and 344SQR tumor models when the triple combination was used. By stimulating the activation of multiple immune pathways within the unirradiated tumor microenvironment, increasing the number of CD8+ T cells and modifying the T cell receptor repertoire in the 344SQR tumor model, NBTXR3 altered the immune microenvironment of the unirradiated tumors. If NBTXR3 can evoke such consistent abscopal effects in both anti-PD1-sensitive as well as anti-PD1-resistant lung cancers, the probability of using it for the treatment of metastatic lung cancer is high regardless of the sensitivity to immunotherapy [120]. Cancer cells can be selectively sensitized to radiotherapy with nanoparticle agents. DM1 was nitrosylated with DM1-NO and then loaded inside poly(lactide-co-glycolic)-block-poly(ethylene glycol) (PLGA-b-PEG) nanoparticles. Through enhanced permeability and retention, nanoparticle encapsulation and nitrosylation suppressed the toxicity of DM1, permitting the drug to reach tumors more efficiently. As a result of irradiation, tumors had an elevated oxidative stress level, triggering the S−N bond cleavage, and the DM1 as well as nitric oxide (NO) were released. As a result of DM1 inhibition, more radiosensitive cells were enriched at the G2/M phase. Peroxynitrites, formed when NO was irradiated, were highly toxic radicals that suppressed tumor growth. The clonogenic assays executed in vitro and in vivo tumor-bearing mice demonstrated that the two components worked synergistically to enhance radiotherapy outcomes [121].

A new clinical option for killing cancer cells is photodynamic therapy (PDT), which can generate cytotoxic reactive oxygen species (ROS). A photosensitizer (PS) drug, light, and oxygen are the three fundamental components of photodynamic therapy (PDT). PS drugs accumulate within tumor sites passively or actively, and when exposed to a specific wavelength of light, they release reactive oxygen species (ROS), which destroy tumor cells. For ROS generation to be effective, tumor cells must accumulate PS before ROS generation can cause tumor destruction. In PDT cancer drug absorption studies, PS selective/targeted uptake and delivery into tumor cells were critical [122,123]. A limitation of PDT is the depth of the visible light’s penetration into tissue. By bombarding the nanoparticles with high penetrating energies of ionizing radiation, researchers could produce large quantities of ROS inside the cells with a ROS-enhanced nanoparticle, hafnium doped hydroxyapatite (Hf:HAp). Ionizing radiation’s impact on Hf:HAp nanoparticles was assessed by means of an in vitro and in vivo model using the A549 cell line. As a result of γ ray exposure, Hf:HAp could produce ROS significantly in cells as determined by the 20,70-dichlorofluorescein diacetate (DCFH-DA) results. According to both the WST-1 and LDH assays, A549 lung cancer cell lines were damaged by changes in ROS levels. By bombarding Hf:HAp nanoparticles with ionizing radiation, in vivo studies demonstrated that tumor growth was inhibited and apoptosis was induced. Using Hf:HAp nanoparticles as a palliative treatment after lung surgery can demonstrate its potential in treating tumors. This acted as a new therapeutic method of interacting with ionizing radiation [124]. Porphyrin HDL nanoparticles developed by researchers possess porphyrin molecules at high density and can dissociate after accumulation in tumor cells very rapidly. This is an image-guided photodynamic therapy (PDT) activatable photosensitizer. Researchers presented a first step in the direction of developing a minimally invasive treatment strategy for peripheral lung cancer and metastatic lymph nodes of advanced lung cancer by using nanoparticles targeting the scavenger receptor class B type I (SR-BI) that is expressed on the lung cancer cells. In human lung cancer cell line H460, porphyrin HDL promoted proper intracellular uptake. Porphyrin HDL produced significant therapeutic effects in vitro after being irradiated with a 671 nm PDT laser. As a result of systemic administration in mice with orthotopic lung cancer xenografts, porphyrin HDL selectively accumulated and photoactivated in tumors, enhancing the contrast between diseased and normal tissues significantly. Furthermore, porphyrin HDL-PDT induced apoptosis in lung tumor cells (73.2%) significantly, without causing toxicity to normal tissues or damaging vital structures adjacent to the lung tumors. Porphyrin HDL-mediated PDT targeted by SR-BI was both selective and effective in vivo and in vitro in treating lung cancer [125].

## 8. Use of Nanoparticles to Reduce the Risk of Normal Tissue Toxicity during Chemoradiation Therapy

Rapid developments in nanomedicine have changed how cancer is diagnosed and treated in recent years. A nanoparticle is a solid colloidal particle with a relatively small size (diameter between 10 and 200 nanometers). With their large surface area to volume ratio, nanoparticles are able to adsorb and contain a variety of anticancer agents, including chemotherapeutic drugs, proteins, DNA, etc. In comparison to the direct use of chemotherapy drugs, NPs have many advantages in delivering chemotherapy drugs. These advantages are given below:

(i) The solubility of chemotherapeutic drugs is improved by using nanoparticles, and they also become stable in vivo.
(ii) Nanoparticle-delivered chemotherapeutic drugs can be administered intravenously to increase biodistribution, extend circulation time, and decrease the adverse effects of reactions to chemotherapy.
(iii) Since solid tumors have different internal dissection characteristics compared to normal healthy tissues, nanocarriers can be used to deliver chemotherapeutic drugs preferentially to the tumor site because of their enhanced permeability and retention (EPR) effects.
(iv) As a result of the tortuous and abnormal nature of angiogenesis in tumors, most lymphatic vessels inside the tumors are compressed and folded with a gap size of 100 nm–2 μm. There is a pressure difference between the tissue at the center of the solid tumor and the tissue around it as a result of the valves that leak and the poorly functioning lymphatic drainage system inside the solid tumor. Due to this difference in pressure in the tumor, molecules with a size between 10 nm and 200 nm accumulate more efficiently and remain there for a long time. This EPR effect helps the nanocarriers to passively target the tumors by retaining their contents several times longer than unpackaged drugs. This is because the retention time of drugs contained in the nanoparticles is about 10 times as long as the retention time of unpackaged drugs [126].
(v) Stimuli-responsive drug delivery can also be executed for the delivery of cancer drugs specifically into the tumor tissues. It is known that the tumor microenvironment is acidic in nature, with a pH of nearly 6.3, whereas the normal tissue surroundings have a physiological pH of 7.2. When a chemotherapeutic drug is encapsulated in a nanostructure that will open up and release the drug at acidic pH, the drug is released near the tumor tissue, not in the normal tissues. Thus, targeted drug delivery can be executed without harming the normal cells. A similar strategy was also applied for delivering radiosensitizers, thereby targeting the cancerous tumors and not the benign cells [127,128].

Hyperthermia can be induced using iron oxide nanoparticles delivered specifically to the cancer tumor by subjecting the animal-bearing tumor to magnetic resonance imaging (MRI). It is known that cancer cells cannot withstand higher temperatures compared to normal cells. In a DLA ascitic tumor induced in mice, polymer-entrapped iron oxide nanoparticles that were surface functionalized with folic acid and chemotherapeutic drugs (5 fluorouracil (5-FU), doxorubicin (DOX), and methotrexate (MTX)) were individually delivered at the liquid tumor by the EPR effect. The animals were subjected to MRI, which allowed the magnetic nanoparticles to vibrate at the tumor site, thereby inducing high heat. Moreover, the drugs were also released inside the tumor since they were entrapped in a polymer matrix that released the drug inside the tumor. Thus, with a combination of chemotherapeutic drugs and hyperthermia, the tumor cells were killed without affecting the normal cells [129]. The tremendous advantages of nanotechnology and nano-enabled drug delivery have shown that specific cancer cells can be targeted and treated with chemotherapeutic drugs or radiation sensitization, thereby protecting the normal cells.

## 9. Second Primary Cancers after Radiotherapy

Radiation-induced second malignancies (RISMs) are an important late side effect of radiation therapy that affects the optimal decision-making process when choosing a treatment option for a patient. The development of RISMs is influenced by a number of factors, such as the age of the individual at the time of radiation, the dose and volume of the irradiated area, the type of organ or tissue irradiated, the radiation technique, and any family history of cancer. There is no known mechanism for RISMs. In oncology, RISMs are becoming increasingly important due to the increased number of cancer survivors, and efforts are being made to decrease or prevent their incidence. Researchers have previously discussed the pathogenesis of RISMs, the factors that contribute to RISMs, as well as screening and prevention strategies for the disease. Researchers have discussed four groups of factors, including (1) pathogenesis (lifestyle and environment, genetic susceptibility, and treatment, such as RT and chemotherapy); (2) aspects such as gender, age, temporal association, RT type, and RT technique; (3) site of RT, and (4) prevention and screening (screening for second malignant neoplasms (SMNs) as well as an intervention to reduce the risk of SMNs [130]. Second primary malignancy (SPM) refers to the development of second cancer after a lung cancer diagnosis at least 5 years earlier. To compare the risk of SPM, age- and propensity score matching (PSM)-adjusted competing risk analyses were assessed. In a previous study, initial lung cancer was treated with radiotherapy in 9162 (19.1%) of 47,911 patients. Second primary melanoma, breast, prostate, thyroid gland, and esophageal cancer rates decreased in patients who received radiation treatment for the initial lung cancer but increased in patients who received radiotherapy for the second primary esophageal cancer. There is no evidence to suggest that radiation therapy for initial lung cancer increases the risk of SPM [131]. On the other hand, radiation-induced lung cancer (LC) has been linked to adjuvant radiotherapy (RT) for breast cancer (BC). From 1992 to 2012, 52,300 women treated for BC and 253,796 age-matched women without BC were included in a population-based cohort study that assessed the risk of primary LC. By using the Kaplan–Meier method, the researchers calculated the cumulative incidence of LC, and they estimated the risk of LC after BC treatment using Cox proportional hazards regressions. BC patients who received RT had a higher cumulative incidence of LC than BC patients who did not receive RT and women who did not have BC. Breathing adaptation techniques, which lower incidental lung doses, may be able to reduce this risk [132]. These findings suggested that the risk of developing second primary cancer after radiation therapy used to cure other cancers is becoming a challenging issue in lung cancer management because it is unknown what factors are responsible for eliciting such an effect.

## 10. Conclusions and Future Perspectives

Repurposing of drugs has now become a strategy to treat several cancers [133]. Lung cancer is among the most unpredictable of cancers throughout the world, and the progression of the disease is very uncertain. The two types of lung cancer, SCLC and NSCLC, are treated with several regimes of chemotherapy or radiation therapy, as well as combinations of chemo and radiation. Other treatment strategies include immunotherapy, gene therapy, etc., all of which possess their own limitations. Recent developments in nanotechnology have opened an avenue for researchers to develop nano-encapsulated chemotherapeutic drugs, radiosensitizers, and drug delivery systems that reduce the disadvantages of conventional therapies. Improving the bioavailability of a drug allows less of the drug to be required for treatment, thereby reducing the associated side effects. Targeted drug delivery or radiosensitizer delivery ensures tumor cell killing, thereby protecting normal cell mortality. The EPR effect improves the retention of the drug/radiosensitizer inside the tumor, thereby eliciting a prolonged effect in tumor cells. Stimuli-responsive drug delivery makes the nanostructured carrier release the drug within the tumor microenvironment instead of normal tissues, thereby protecting the normal cells. There are only a few FDA-approved nanoformulated chemotherapeutic drugs available in the market that are used for chemoradiotherapy. Several nanoparticles have been shown to improve the radiation effect in tumors with high Z values and are non-toxic. Gold, silver, and platinum are a few of the nanoparticles that have been shown to specifically kill the tumor cells by enhancing radiation efficiency. The nanoparticles can also be functionalized using different molecules to further improve the tumor killing effect. Further research is necessary to develop more nano-formulated drugs that can be used for clinical trials.

## Figures and Tables

**Table 1:** Nanoformulated chemotherapeutic drugs that are used for lung cancer or undergoing clinical trials (reproduced from [103]).

Nanomaterials Used	Drugs Formulated	Company	Disease Indications	Route	Status
Liposomes	Dox	Ortho Biotech	Antineoplastic	IV	Approved/1995
	Daunorubicin	Diatos	Antineoplastic	IV	Approved/1996
	Cisplatin	Alza	Lung cancer	IV	Phase-III trail
	Paclitaxel	Neopharma	Lung cancer	IV	Phase-II trial
	BLP 25		Lung cancer	IV	Phase-II trial
Polymer	Interferon α-2a	Genentech	Hepatitis C	IV	Approved/2002
	Interferon α-2b	Merck	Hepatitis C	IV	Approved/2001
	Leuprolide acetate	Fierce Parma	Prostate cancer	IV	Approved/2002
	Dox		Breast cancer, adenocarcinoma of the esophagus	IV	Phase-III/I trial
Metallic	Ferumoxtran-10	Advanced Mag.	Tumor imaging	IV	Filed
	TNF-α	Cyt Immune Sci.	Solid tumor	IV	Phase-I trial
	Porfimer	Concordia Labs	Lung cancer	IV	Approved/1995
	Dox	-	Solid tumor	IV	Phase-I trial
	Paclitaxel	-	Gastric and colon cancer	IV	Phase-I trial

**Table 2:** Summarizes the improvement of radiation therapy with nanoparticles in vitro lung cancer models/cells.

Nanoparticle Used	Cell Line/Cancer Model Used	Summary of the Study	Reference
Cisplatin nanoparticles (CNPs), gold nanoparticles, (GNPs), and carboplatin nanoparticles (CBNPs)	An analytical method was used to estimate the dose enhancement to lung tumors due to radiation-induced photoelectrons generated by NPs administered via inhalation (IR) versus intravenous (IV) administration, based on Monte Carlo-generated megavoltage energy spectra. In this model, the tumor voxel is sized 10 μm × 10 μm × 10 μm. It was assumed that nanoparticles were distributed evenly within the tumor subvolume, as indicated by this model. Under the conditions of different drug concentrations, the number of nanoparticles or concentrations was determined.	A range of nanoparticle concentrations and tumor sizes were considered in order to calculate the dose enhancement factor (DEF), which was defined as the ratio of the radiotherapy dose with and without nanoparticles. A comparison was then made between the DEF for IR and IV. The results of these experimental studies indicated that IR could deliver 3.5–14.6 times higher NP concentrations to the lungs than IV. Based on the results of this study, IR administration of targeted high-Z CNPs/CBNPs/GNPs could significantly reduce lung tumor growth compared with IV administration during external beam radiotherapy.	[110]
Iron oxide nanoparticles, TAT (transactivator of transcription)-conjugated iron oxide nanoparticles	A549 cells (lung cancer cells)	A cell penetrating peptide, TAT, was conjugated to iron oxide nanoparticles in this project to escape lysosomal encapsulation after internalization by cancer cells and catalyze hydroxyl radical production. A TAT functionalized iron oxide nanoparticle as well as an uncoated iron oxide nanoparticle permeabilized lysosomal membranes. TAT-functionalized nanoparticles and radiation also compromised mitochondrial integrity in A549 cells. A significant increase in ROS generation was also observed when TAT-functionalized nanoparticles were pre-treated with radiation. The combination of TAT-functionalized nanoparticles and radiation had a synergistic effect on long-term viability. Since the nanoparticles alone did not cause significant toxicity, it is likely that TAT functionalized nanoparticles sensitized the cells to radiation therapy.	[111]
Apigenin stabilized gold nanoparticles (AuNPs)	A549 cells (lung cancer cells)	In this study, gold nanoparticles stabilized by apigenin were used for in vitro cancer treatment with chemotherapy and enhanced radiotherapy. Cell apoptosis, proliferation inhibition, and arrest in G0/G1 phases were observed as a result of nanoparticle interaction with lung cancer cells (A549). In a study using X-rays and nanoparticles together, it was found to generate an additive anti-cancer effect as a result of the chemotherapeutic functions of apigenin, as well as the enhanced radiation killing effect caused by the interaction between the nanoparticles and the X-rays.	[112]
Selenium nanoparticles (SeNPs)	A549 cells (lung cancer cells) and IMR-90 normal fibroblast cells	Biosynthesized and characterized selenium nanoparticles were applied to the treatment of cancer cells (A549 cells) and normal cells (IMR-90 cells). Under the influence of X-rays, selenium nanoparticles were tested for their radio-sensitizing effect against cancer as well as healthy cells. A combination of SeNPs and X-rays was found to be cytotoxic to lung cancer cells in this study.	[113]
A folate receptor-targeting multifunctional dual drug-loaded nanoparticle (MDNP) containing a poly(N-isopropylacrylamide)-carboxymethyl chitosan shell and poly lactic-coglycolic acid (PLGA) core	A549 and H460 lung cancer cells	To treat lung cancer effectively, researchers developed a multifunctional dual drug-loaded nanoparticle (MDNP) that targets folate receptors with a poly(N-isopropylacrylamide)-carboxymethyl chitosan shell and polylactic-coglycolic acid (PLGA) core containing a poly(N-isopropylacrylamide)-carboxymethyl chitosan shell. This formulation provided controlled release of the encapsulated radiosensitizer NU7441 and the FDA-approved chemotherapy drug gemcitabine, which is used in lung cancer chemoradiation therapy. According to these results, MDNPs have the potential to be used as nanovehicles for the chemoradiation sensitization of lung cancer at the same time.	[114]
Mesoporous silica nanoparticle (MSNP) with surface functionalization.	NSCLC cells A549 (CCL-185) and H460 (HTB-177)	The researchers developed a new targeted therapy for NSCLC based on cetuximab-conjugated nanoparticles that deliver small interfering RNA (siRNA) against polo-like kinase 1 (PLK1). PLK1 is a key mitotic regulator whose inhibition improves radiation sensitivity, while EGFR is overexpressed in 50% of lung cancer patients. In this study, a nanoparticle construct called C-siPLK1-NP was used to target EGFR+ NSCLC cells and reduce the expression of PLK1, resulting in cell death and G2/M arrest. They found that C-siPLK1-NP is an effective targeted therapy as well as a potent radiation sensitizer for NSCLC.	[115]
Gadolinium-based nanoparticle, AGuIX	H1299 NSCLC cell line	An efficient radiosensitizer based on gadolinium, called AGuIX, was developed for magnetic resonance imaging-guided radiotherapy. It appears that low-energy photoelectrons and Auger electron interactions are responsible for the amplified radiation effects of AGuIX nanoparticles. This study in H1299 NSCLC cells demonstrated that AGuIX nanoparticles enhanced radiation-induced DNA double-strand breaks and slowed DNA repair. In addition, researchers found that the AGuIX nanoparticles significantly exacerbated tumor cell damage, under radiation therapy, in an H1299 mouse xenograft model.	[116]
Selenium nanoparticles (nano-Se)	A549 and NCI-H23 cells	A549 and NCI-H23 cells were treated with selenium nanoparticles (nano-Se) and radiotherapy to study the effects on proliferation, invasion, migration, and apoptosis. Increased nano-Se concentration increased the uptake of nano-Se in lung cancer cells. In combination with radiotherapy, nano-Se decreased the proliferation activity of NSCLC cell lines A549 and NCI-H23 (all *p* < 0.05). The outcome of this study indicated that nano-Se might also be used in clinical lung cancer treatment as a radiosensitizer.	[117]

## Data Availability

Not applicable.

## References

[1] Siegel R.L., Miller K.D., Fuchs H.E., Jemal A. (2022). Cancer statistics, 2022. CA A Cancer J. Clin..

[2] Doumat G., Daher D., Zerdan M.B., Nasra N., Bahmad H.F., Recine M., Poppiti R. (2023). Drug Repurposing in Non-Small Cell Lung Carcinoma: Old Solutions for New Problems. Curr. Oncol..

[3] Pallavi P., Harini K., Crowder S., Ghosh D., Gowtham P., Girigoswami K., Girigoswami A. (2023). Rhodamine-Conjugated Anti-Stokes Gold Nanoparticles with Higher ROS Quantum Yield as Theranostic Probe to Arrest Cancer and MDR Bacteria. Appl. Biochem. Biotechnol..

[4] Gowtham P., Girigoswami K., Pallavi P., Harini K., Gurubharath I., Girigoswami A. (2022). Alginate-Derivative Encapsulated Carbon Coated Manganese-Ferrite Nanodots for Multimodal Medical Imaging. Pharmaceutics.

[5] Soni A., Bhandari M.P., Tripathi G.K., Bundela P., Khiriya P.K., Khare P.S., Kashyap M.K., Dey A., Vellingiri B., Sundaramurthy S. (2023). Nano-biotechnology in tumour and cancerous disease: A perspective review. J. Cell. Mol. Med..

[6] Jagdale Swati C., HableAsawaree A., ChabukswarAnuruddha R. (2023). Nanomedicine in lung cancer therapy. Adv. Nov. Formul. Drug Deliv..

[7] Ou W., Stewart S., White A., Kwizera E.A., Xu J., Fang Y., Shamul J.G., Xie C., Nurudeen S., Tirada N.P. (2023). In-situ cryo-immune engineering of tumor microenvironment with cold-responsive nanotechnology for cancer immunotherapy. Nature Commun..

[8] García-Fernández C., Fornaguera C., Borrós S. (2020). Nanomedicine in non-small cell lung cancer: From conventional treatments to immunotherapy. Cancers.

[9] Forde P.M., Spicer J., Lu S., Provencio M., Mitsudomi T., Awad M.M., Felip E., Broderick S.R., Brahmer J.R., Swanson S.J. (2022). Neoadjuvant nivolumab plus chemotherapy in resectable lung cancer. N. Engl. J. Med..

[10] Paz-Ares L., Ciuleanu T.-E., Cobo M., Schenker M., Zurawski B., Menezes J., Richardet E., Bennouna J., Felip E., Juan-Vidal O. (2021). First-line nivolumab plus ipilimumab combined with two cycles of chemotherapy in patients with non-small-cell lung cancer (CheckMate 9LA): An international, randomised, open-label, phase 3 trial. Lancet Oncol..

[11] Wang J., Lu S., Yu X., Hu Y., Sun Y., Wang Z., Zhao J., Yu Y., Hu C., Yang K. (2021). Tislelizumab plus chemotherapy vs chemotherapy alone as first-line treatment for advanced squamous non–small-cell lung cancer: A phase 3 randomized clinical trial. JAMA Oncol..

[12] West H., McCleod M., Hussein M., Morabito A., Rittmeyer A., Conter H.J., Kopp H.-G., Daniel D., McCune S., Mekhail T. (2019). Atezolizumab in combination with carboplatin plus nab-paclitaxel chemotherapy compared with chemotherapy alone as first-line treatment for metastatic non-squamous non-small-cell lung cancer (IMpower130): A multicentre, randomised, open-label, phase 3 trial. Lancet Oncol..

[13] Shu C.A., Gainor J.F., Awad M.M., Chiuzan C., Grigg C.M., Pabani A., Garofano R.F., Stoopler M.B., Cheng S.K., White A. (2020). Neoadjuvant atezolizumab and chemotherapy in patients with resectable non-small-cell lung cancer: An open-label, multicentre, single-arm, phase 2 trial. Lancet Oncol..

[14] Rizvi N.A., Cho B.C., Reinmuth N., Lee K.H., Luft A., Ahn M.-J., van den Heuvel M.M., Cobo M., Vicente D., Smolin A. (2020). Durvalumab with or without tremelimumab vs standard chemotherapy in first-line treatment of metastatic non–small cell lung cancer: The MYSTIC phase 3 randomized clinical trial. JAMA Oncol..

[15] Reck M., Ciuleanu T.-E., Dols M.C., Schenker M., Zurawski B., Menezes J., Richardet E., Bennouna J., Felip E., Juan-Vidal O. (2020). Nivolumab (NIVO)+ ipilimumab (IPI)+ 2 cycles of platinum-doublet chemotherapy (chemo) vs 4 cycles chemo as first-line (1L) treatment (tx) for stage IV/recurrent non-small cell lung cancer (NSCLC): CheckMate 9LA. J. Clin. Oncol..

[16] Reck M., Rodríguez-Abreu D., Robinson A.G., Hui R., Csőszi T., Fülöp A., Gottfried M., Peled N., Tafreshi A., Cuffe S. (2021). Five-year outcomes with pembrolizumab versus chemotherapy for metastatic non–small-cell lung cancer with PD-L1 tumor proportion score ≥ 50%. J. Clin. Oncol..

[17] Provencio M., Nadal E., Insa A., García-Campelo M.R., Casal-Rubio J., Dómine M., Majem M., Rodríguez-Abreu D., Martínez-Martí A., Carpeño J.D.C. (2020). Neoadjuvant chemotherapy and nivolumab in resectable non-small-cell lung cancer (NADIM): An open-label, multicentre, single-arm, phase 2 trial. Lancet Oncol..

[18] Noronha V., Patil V.M., Joshi A., Menon N., Chougule A., Mahajan A., Janu A., Purandare N., Kumar R., More S. (2020). Gefitinib versus gefitinib plus pemetrexed and carboplatin chemotherapy in EGFR-mutated lung cancer. J. Clin. Oncol..

[19] Hosomi Y., Morita S., Sugawara S., Kato T., Fukuhara T., Gemma A., Takahashi K., Fujita Y., Harada T., Minato K. (2020). Gefitinib alone versus gefitinib plus chemotherapy for non–small-cell lung cancer with mutated epidermal growth factor receptor: NEJ009 study. J. Clin. Oncol..

[20] Kris M.G., Gaspar L.E., Chaft J.E., Kennedy E.B., Azzoli C.G., Ellis P.M., Lin S.H., Pass H.I., Seth R., Shepherd F.A. (2017). Adjuvant Systemic Therapy and Adjuvant Radiation Therapy for Stage I to IIIA Completely Resected Non–Small-Cell Lung Cancers: American Society of Clinical Oncology/Cancer Care Ontario Clinical Practice Guideline Update. J. Clin. Oncol..

[21] Simone II C.B., Bogart J.A., Cabrera A.R., Daly M.E., DeNunzio N.J., Detterbeck F., Faivre-Finn C., Gatschet N., Gore E., Jabbour S.K. (2020). Radiation therapy for small cell lung cancer: An ASTRO clinical practice guideline. Radiat. Oncol..

[22] Vinod S.K., Hau E. (2020). Radiotherapy treatment for lung cancer: Current status and future directions. Respirology.

[23] Videtic G.M., Paulus R., Singh A.K., Chang J.Y., Parker W., Olivier K.R., Timmerman R.D., Komaki R.R., Urbanic J.J., Stephans K.L. (2019). Long-term follow-up on NRG Oncology RTOG 0915 (NCCTG N0927): A randomized phase 2 study comparing 2 stereotactic body radiation therapy schedules for medically inoperable patients with stage I peripheral non-small cell lung cancer. Int. J. Radiat. Oncol. Biol. Phys..

[24] Prezzano K.M., Ma S.J., Hermann G.M., Rivers C.I., Gomez-Suescun J.A., Singh A.K. (2019). Stereotactic body radiation therapy for non-small cell lung cancer: A review. World J. Clin. Oncol..

[25] Cerullo M., Lee H.-J., Kelsey C., Farrow N.E., Scales C.D., Tong B.C. (2023). Surgical evaluation in patients undergoing radiation therapy for early-stage lung cancer. Ann. Thorac. Surg..

[26] Singh A.K., Gomez-Suescun J.A., Stephans K.L., Bogart J.A., Hermann G.M., Tian L., Groman A., Videtic G.M. (2019). One versus three fractions of stereotactic body radiation therapy for peripheral stage I to II non-small cell lung cancer: A randomized, multi-institution, phase 2 trial. Int. J. Radiat. Oncol. Biol. Phys..

[27] Ladbury C.J., Rusthoven C.G., Camidge D.R., Kavanagh B.D., Nath S.K. (2019). Impact of radiation dose to the host immune system on tumor control and survival for stage III non-small cell lung cancer treated with definitive radiation therapy. Int. J. Radiat. Oncol. Biol. Phys..

[28] D’Andrea M.A., Reddy G.K. (2021). Systemic effects of radiation therapy-induced abscopal responses in patients with advanced lung cancer. Oncology.

[29] Finazzi T., Palacios M.A., Haasbeek C.J., Admiraal M.A., Spoelstra F.O., Bruynzeel A.M., Slotman B.J., Lagerwaard F.J., Senan S. (2020). Stereotactic MR-guided adaptive radiation therapy for peripheral lung tumors. Radiother. Oncol..

[30] Haslett K., Bayman N., Franks K., Groom N., Harden S.V., Harris C., Hanna G., Harrow S., Hatton M., McCloskey P. (2021). Isotoxic intensity modulated radiation therapy in stage III non-small cell lung cancer: A feasibility study. Int. J. Radiat. Oncol. Biol. Phys..

[31] Or M., Liu B., Lam J., Vinod S., Xuan W., Yeghiaian-Alvandi R., Hau E. (2021). A systematic review and meta-analysis of treatment-related toxicities of curative and palliative radiation therapy in non-small cell lung cancer. Sci. Rep..

[32] Wang K., Tepper J.E. (2021). Radiation therapy-associated toxicity: Etiology, management, and prevention. CA A Cancer J. Clin..

[33] Conibear J., Limited A.U. (2020). Rationale for concurrent chemoradiotherapy for patients with stage III non-small-cell lung cancer. Br. J. Cancer.

[34] Borghetti P., Branz J., Volpi G., Pancera S., Buraschi R., Bianchi L.N.C., Bonù M.L., Greco D., Facheris G., Tomasi C. (2022). Home-based pulmonary rehabilitation in patients undergoing (chemo) radiation therapy for unresectable lung cancer: A prospective explorative study. Radiol. Med..

[35] Diamond B.H., Verma N., Shukla U.C., Park H.S., Koffer P.P. (2022). Consolidative thoracic radiation therapy after first-line chemotherapy and immunotherapy in extensive-stage small cell lung cancer: A multi-institutional case series. Adv. Radiat. Oncol..

[36] Kubicek G.J., Khrizman P., Squillante C., Callahan K., Xu Q., Abouzgheib W., Boujaoude Z., Patel A., Hageboutros A. (2022). Stereotactic body radiotherapy and systemic dose chemotherapy for locally advanced lung cancer: Single arm phase 2 study. Am. J. Clin. Oncol..

[37] Mamdani H., Induru R., Jalal S.I. (2015). Novel therapies in small cell lung cancer. Transl. Lung Cancer Res..

[38] Leonetti A., Wever B., Mazzaschi G., Assaraf Y.G., Rolfo C., Quaini F., Tiseo M., Giovannetti E. (2019). Molecular basis and rationale for combining immune checkpoint inhibitors with chemotherapy in non-small cell lung cancer. Drug Resist. Updates.

[39] Yang K., Li J., Bai C., Sun Z., Zhao L. (2020). Efficacy of immune checkpoint inhibitors in non-small-cell lung cancer patients with different metastatic sites: A systematic review and meta-analysis. Front. Oncol..

[40] Ferrara R., Imbimbo M., Malouf R., Paget-Bailly S., Calais F., Marchal C., Westeel V. (2020). Single or combined immune checkpoint inhibitors compared to first-line platinum-based chemotherapy with or without bevacizumab for people with advanced non-small cell lung cancer. Cochrane Database Syst. Rev..

[41] Soh J., Hamada A., Fujino T., Mitsudomi T. (2021). Perioperative therapy for non-small cell lung cancer with immune checkpoint inhibitors. Cancers.

[42] Dafni U., Tsourti Z., Vervita K., Peters S. (2019). Immune checkpoint inhibitors, alone or in combination with chemotherapy, as first-line treatment for advanced non-small cell lung cancer. A systematic review and network meta-analysis. Lung Cancer.

[43] Blumenthal G.M., Zhang L., Zhang H., Kazandjian D., Khozin S., Tang S., Goldberg K., Sridhara R., Keegan P., Pazdur R. (2017). Milestone analyses of immune checkpoint inhibitors, targeted therapy, and conventional therapy in metastatic non–small cell lung cancer trials: A meta-analysis. JAMA Oncol..

[44] Swisher S.G., Roth J.A., Carbone D.P. (2002). Genetic and immunologic therapies for lung cancer. Semin. Oncol..

[45] Toloza E.M., Morse M.A., Lyerly H.K. (2006). Gene therapy for lung cancer. J. Cell. Biochem..

[46] Lara-Guerra H., Roth J.A. (2016). Gene therapy for lung cancer. Crit. Rev. Oncog..

[47] Prabavathy D., Swarnalatha Y., Ramadoss N. (2018). Lung cancer stem cells—Origin, characteristics and therapy. Stem Cell Investig..

[48] Xin Y.l., Xue F.z., Ge B.s., Zhao F.r., Shi B., Zhang W. (1997). Electrochemical treatment of lung cancer. Bioelectromagn. J. Bioelectromagn. Soc. Soc. Phys. Regul. Biol. Med. Eur. Bioelectromagn. Assoc..

[49] Xue W., Dahlman J.E., Tammela T., Khan O.F., Sood S., Dave A., Cai W., Chirino L.M., Yang G.R., Bronson R. (2014). Small RNA combination therapy for lung cancer. Proc. Natl. Acad. Sci. USA.

[50] Xu C., Tian H., Wang P., Wang Y., Chen X. (2016). The suppression of metastatic lung cancer by pulmonary administration of polymer nanoparticles for co-delivery of doxorubicin and Survivin siRNA. Biomater. Sci..

[51] Dupuy D.E., DiPetrillo T., Gandhi S., Ready N., Ng T., Donat W., Mayo-Smith W.W. (2006). Radiofrequency ablation followed by conventional radiotherapy for medically inoperable stage I non-small cell lung cancer. Chest.

[52] Rivera Díaz M., Vivas-Mejia P.E. (2013). Nanoparticles as drug delivery systems in cancer medicine: Emphasis on RNAi-containing nanoliposomes. Pharmaceuticals.

[53] Mottaghitalab F., Farokhi M., Fatahi Y., Atyabi F., Dinarvand R. (2019). New insights into designing hybrid nanoparticles for lung cancer: Diagnosis and treatment. J. Control. Release.

[54] Gholami L., Ivari J.R., Nasab N.K., Oskuee R.K., Sathyapalan T., Sahebkar A. (2023). Recent advances in lung cancer therapy based on nanomaterials: A review. Curr. Med. Chem..

[55] Li G., Liu D., Zuo Y.Y. (2022). Nano-bio Interactions in the Lung. Nanomedicine.

[56] Smith L., Byrne H.L., Waddington D., Kuncic Z. (2022). Nanoparticles for MRI-guided radiation therapy: A review. Cancer Nanotechnol..

[57] Sharmiladevi P., Akhtar N., Haribabu V., Girigoswami K., Chattopadhyay S., Girigoswami A.J.N.-S. (2019). Excitation wavelength independent carbon-decorated ferrite nanodots for multimodal diagnosis and stimuli responsive therapy. ACS Appl. Bio Mater..

[58] Sharma U., Jagannathan N.R. (2022). Magnetic Resonance Imaging (MRI) and MR Spectroscopic Methods in Understanding Breast Cancer Biology and Metabolism. Metabolites.

[59] Haribabu V., Sharmiladevi P., Akhtar N., Farook A.S., Girigoswami K., Girigoswami A.J.N.-S. (2019). Label free ultrasmall fluoromagnetic ferrite-clusters for targeted cancer imaging and drug delivery. Curr. Drug Deliv..

[60] Matsumura Y., Maeda H. (1986). A new concept for macromolecular therapeutics in cancer chemotherapy: Mechanism of tumoritropic accumulation of proteins and the antitumor agent smancs. Cancer Res..

[61] Wang A.Z., Langer R., Farokhzad O.C. (2012). Nanoparticle delivery of cancer drugs. Annu. Rev. Med..

[62] Elbashir S.M., Harborth J., Lendeckel W., Yalcin A., Weber K., Tuschl T. (2001). Duplexes of 21-nucleotide RNAs mediate RNA interference in cultured mammalian cells. Nature.

[63] Zamore P.D., Tuschl T., Sharp P.A., Bartel D.P. (2000). RNAi: Double-stranded RNA directs the ATP-dependent cleavage of mRNA at 21 to 23 nucleotide intervals. Cell.

[64] Salem A.K., Patil S.D., Burgess D.J. (2012). Recent progress in non-viral nucleic acids delivery. Int. J. Pharm..

[65] Gencer A., Duraloglu C., Ozbay S., Ciftci T.T., Yabanoglu-Ciftci S., Arica B. (2021). Recent advances in treatment of lung cancer: Nanoparticle-based drug and siRNA delivery systems. Curr. Drug Deliv..

[66] Kim Y.-D., Park T.-E., Singh B., Maharjan S., Choi Y.-J., Choung P.-H., Arote R.B., Cho C.-S. (2015). Nanoparticle-mediated delivery of siRNA for effective lung cancer therapy. Nanomedicine.

[67] Itani R., Al Faraj A. (2019). SiRNA conjugated nanoparticles—A next generation strategy to treat lung cancer. Int. J. Mol. Sci..

[68] Amarzguioui M., Peng Q., Wiiger M.T., Vasovic V., Babaie E., Holen T., Nesland J.M., Prydz H. (2006). Ex vivo and in vivo delivery of anti-tissue factor short interfering RNA inhibits mouse pulmonary metastasis of B16 melanoma cells. Clin. Cancer Res..

[69] Zhang C., Tang N., Liu X., Liang W., Xu W., Torchilin V.P. (2006). siRNA-containing liposomes modified with polyarginine effectively silence the targeted gene. J. Control. Release.

[70] Jiang M., Zhang E., Liang Z., Zhao Y., Zhang S., Xu H., Wang H., Shu X., Kang X., Sun L. (2019). Liposome-based co-delivery of 7-O-geranyl-quercetin and IGF-1R siRNA for the synergistic treatment of non-small cell lung cancer. J. Drug Deliv. Sci. Technol..

[71] Dong Z., Yin Y., Luo J., Li B., Lou F., Wang Q., Zhou Q., Ye B., Wang Y. (2022). An FGFR1-Binding Peptide Modified Liposome for siRNA Delivery in Lung Cancer. Int. J. Mol. Sci..

[72] Zhang C., Zhang S., Zhi D., Zhao Y., Cui S., Cui J. (2020). Co-delivery of paclitaxel and survivin siRNA with cationic liposome for lung cancer therapy. Colloids Surf. A Physicochem. Eng. Asp..

[73] Barenholz Y.C. (2012). Doxil^®^—The first FDA-approved nano-drug: Lessons learned. J. Control. Release.

[74] Petersen G.H., Alzghari S.K., Chee W., Sankari S.S., La-Beck N.M. (2016). Meta-analysis of clinical and preclinical studies comparing the anticancer efficacy of liposomal versus conventional non-liposomal doxorubicin. J. Control. Release.

[75] Mukherjee A., Bhattacharyya J., Sagar M.V., Chaudhuri A. (2013). Liposomally encapsulated CDC20 siRNA inhibits both solid melanoma tumor growth and spontaneous growth of intravenously injected melanoma cells on mouse lung. Drug Deliv. Transl. Res..

[76] Song X.-L., Ju R.-J., Xiao Y., Wang X., Liu S., Fu M., Liu J.-J., Gu L.-Y., Li X.-T., Cheng L. (2017). Application of multifunctional targeting epirubicin liposomes in the treatment of non-small-cell lung cancer. Int. J. Nanomed..

[77] Lee H.-Y., Mohammed K.A., Nasreen N. (2016). Nanoparticle-based targeted gene therapy for lung cancer. Am. J. Cancer Res..

[78] Sukumar U.K., Bhushan B., Dubey P., Matai I., Sachdev A., Packirisamy G. (2013). Emerging applications of nanoparticles for lung cancer diagnosis and therapy. Int. Nano Lett..

[79] Danhier F., Lecouturier N., Vroman B., Jérôme C., Marchand-Brynaert J., Feron O., Préat V. (2009). Paclitaxel-loaded PEGylated PLGA-based nanoparticles: In vitro and in vivo evaluation. J. Control. Release.

[80] Derakhshandeh K., Erfan M., Dadashzadeh S. (2007). Encapsulation of 9-nitrocamptothecin, a novel anticancer drug, in biodegradable nanoparticles: Factorial design, characterization and release kinetics. Eur. J. Pharm. Biopharm..

[81] Braden A.R., Kafka M.T., Cunningham L., Jones H., Vishwanatha J.K. (2009). Polymeric nanoparticles for sustained down-regulation of annexin A2 inhibit prostate tumor growth. J. Nanosci. Nanotechnol..

[82] Ogris M., Wagner E. (2002). Tumor-targeted gene transfer with DNA polyplexes. Somat. Cell Mol. Genet..

[83] Nguyen J., Xie X., Neu M., Dumitrascu R., Reul R., Sitterberg J., Bakowsky U., Schermuly R., Fink L., Schmehl T. (2008). Effects of cell-penetrating peptides and pegylation on transfection efficiency of polyethylenimine in mouse lungs. J. Gene Med. A Cross-Discip. J. Res. Sci. Gene Transf. Its Clin. Appl..

[84] Koshkina N.V., Agoulnik I.Y., Melton S.L., Densmore C.L., Knight V. (2003). Biodistribution and pharmacokinetics of aerosol and intravenously administered DNA–polyethyleneimine complexes: Optimization of pulmonary delivery and retention. Mol. Ther..

[85] Gautam A., Densmore C.L., Melton S., Golunski E., Waldrep J.C. (2002). Aerosol delivery of PEI–p53 complexes inhibits B16-F10 lung metastases through regulation of angiogenesis. Cancer Gene Ther..

[86] Kimura S., Egashira K., Chen L., Nakano K., Iwata E., Miyagawa M., Tsujimoto H., Hara K., Morishita R., Sueishi K. (2009). Nanoparticle-mediated delivery of nuclear factor κB decoy into lungs ameliorates monocrotaline-induced pulmonary arterial hypertension. Hypertension.

[87] Ziady A.-G., Gedeon C.R., Muhammad O., Stillwell V., Oette S.M., Fink T.L., Quan W., Kowalczyk T.H., Hyatt S.L., Payne J. (2003). Minimal toxicity of stabilized compacted DNA nanoparticles in the murine lung. Mol. Ther..

[88] Ziady A.-G., Gedeon C.R., Miller T., Quan W., Payne J.M., Hyatt S.L., Fink T.L., Muhammad O., Oette S., Kowalczyk T. (2003). Transfection of airway epithelium by stable PEGylated poly-L-lysine DNA nanoparticles in vivo. Mol. Ther..

[89] Kaul G., Amiji M. (2005). Tumor-targeted gene delivery using poly (ethylene glycol)-modified gelatin nanoparticles: In vitro and in vivo studies. Pharm. Res..

[90] Issa M.M., Köping-Höggård M., Tømmeraas K., Vårum K.M., Christensen B.E., Strand S.P., Artursson P. (2006). Targeted gene delivery with trisaccharide-substituted chitosan oligomers in vitro and after lung administration in vivo. J. Control. Release.

[91] Almeida A.J., Souto E. (2007). Solid lipid nanoparticles as a drug delivery system for peptides and proteins. Adv. Drug Deliv. Rev..

[92] Beck-Broichsitter M., Gauss J., Packhaeuser C.B., Lahnstein K., Schmehl T., Seeger W., Kissel T., Gessler T. (2009). Pulmonary drug delivery with aerosolizable nanoparticles in an ex vivo lung model. Int. J. Pharm..

[93] Bivas-Benita M., Oudshoorn M., Romeijn S., van Meijgaarden K., Koerten H., van der Meulen H., Lambert G., Ottenhoff T., Benita S., Junginger H. (2004). Cationic submicron emulsions for pulmonary DNA immunization. J. Control. Release.

[94] Hu J., Fu S., Peng Q., Han Y., Xie J., Zan N., Chen Y., Fan J. (2017). Paclitaxel-loaded polymeric nanoparticles combined with chronomodulated chemotherapy on lung cancer: In vitro and in vivo evaluation. Int. J. Pharm..

[95] Wang X., Chen H., Zeng X., Guo W., Jin Y., Wang S., Tian R., Han Y., Guo L., Han J. (2019). Efficient lung cancer-targeted drug delivery via a nanoparticle/MSC system. Acta Pharm. Sin. B.

[96] Sivarajakumar R., Mallukaraj D., Kadavakollu M., Neelakandan N., Chandran S., Bhojaraj S., Karri V.V.S.R. (2018). Nanoparticles for the treatment of lung cancers. J. Young Pharm..

[97] Perepelyuk M., Sacko K., Thangavel K., Shoyele S.A. (2018). Evaluation of MUC1-aptamer functionalized hybrid nanoparticles for targeted delivery of miRNA-29b to nonsmall cell lung cancer. Mol. Pharm..

[98] Rizvi N.A., Riely G.J., Azzoli C.G., Miller V.A., Ng K.K., Fiore J., Chia G., Brower M., Heelan R., Hawkins M.J. (2008). Phase I/II trial of weekly intravenous 130-nm albumin-bound paclitaxel as initial chemotherapy in patients with stage IV non–small-cell lung cancer. J. Clin. Oncol..

[99] Mukherjee A., Paul M., Mukherjee S. (2019). Recent progress in the theranostics application of nanomedicine in lung cancer. Cancers.

[100] Knights O.B., McLaughlan J.R. (2018). Gold nanorods for light-based lung cancer theranostics. Int. J. Mol. Sci..

[101] Silva C.O., Pinho J.O., Lopes J.M., Almeida A.J., Gaspar M.M., Reis C. (2019). Current trends in cancer nanotheranostics: Metallic, polymeric, and lipid-based systems. Pharmaceutics.

[102] Sarkar S., Osama K., Mohammad Sajid Jamal Q., Amjad Kamal M., Sayeed U., Khan K.A., Siddiqui H., Akhtar S. (2017). Advances and implications in nanotechnology for lung cancer management. Curr. Drug Metab..

[103] Madni A., Batool A., Noreen S., Maqbool I., Rehman F., Kashif P.M., Tahir N., Raza A. (2017). Novel nanoparticulate systems for lung cancer therapy: An updated review. J. Drug Target..

[104] Kwatra D., Venugopal A., Anant S. (2013). Nanoparticles in radiation therapy: A summary of various approaches to enhance radiosensitization in cancer. Transl. Cancer Res..

[105] Pandey A., Vighetto V., Di Marzio N., Ferraro F., Hirsch M., Ferrante N., Mitra S., Grattoni A., Filgueira C.S. (2020). Gold Nanoparticles Radio-Sensitize and Reduce Cell Survival in Lewis Lung Carcinoma. Nanomaterials.

[106] Wang C., Li X., Wang Y., Liu Z., Fu L., Hu L. (2013). Enhancement of radiation effect and increase of apoptosis in lung cancer cells by thio-glucose-bound gold nanoparticles at megavoltage radiation energies. J. Nanoparticle Res..

[107] Zhuang M., Jiang S., Gu A., Chen X., Mingyan E. (2021). Radiosensitizing effect of gold nanoparticle loaded with small interfering RNA-SP1 on lung cancer: AuNPs-si-SP1 regulates GZMB for radiosensitivity. Transl. Oncol..

[108] Boateng F., Ngwa W. (2019). Delivery of nanoparticle-based radiosensitizers for radiotherapy applications. Int. J. Mol. Sci..

[109] Iyer R., Ramachandramoorthy H., Nguyen T., Xu C., Fu H., Kotadia T., Chen B., Hong Y., Saha D., Nguyen K.T. (2022). Lung Cancer Targeted Chemoradiotherapy via Dual-Stimuli Responsive Biodegradable Core-Shell Nanoparticles. Pharmaceutics.

[110] Hao Y., Altundal Y., Moreau M., Sajo E., Kumar R., Ngwa W. (2015). Potential for enhancing external beam radiotherapy for lung cancer using high-Z nanoparticles administered via inhalation. Phys. Med. Biol..

[111] Hauser A.K., Mitov M.I., Daley E.F., McGarry R.C., Anderson K.W., Hilt J.Z. (2016). Targeted iron oxide nanoparticles for the enhancement of radiation therapy. Biomaterials.

[112] Jiang J., Mao Q., Li H., Lou J. (2017). Apigenin stabilized gold nanoparticles increased radiation therapy efficiency in lung cancer cells. Int. J. Clin. Exp. Med..

[113] Cruz L.Y., Wang D., Liu J. (2019). Biosynthesis of selenium nanoparticles, characterization and X-ray induced radiotherapy for the treatment of lung cancer with interstitial lung disease. J. Photochem. Photobiol. B Biol..

[114] Menon J.U., Kuriakose A., Iyer R., Hernandez E., Gandee L., Zhang S., Takahashi M., Zhang Z., Saha D., Nguyen K.T. (2017). Dual-drug containing core-shell nanoparticles for lung cancer therapy. Sci. Rep..

[115] Reda M., Ngamcherdtrakul W., Gu S., Bejan D.S., Siriwon N., Gray J.W., Yantasee W. (2019). PLK1 and EGFR targeted nanoparticle as a radiation sensitizer for non-small cell lung cancer. Cancer Lett..

[116] Du Y., Sun H., Lux F., Xie Y., Du L., Xu C., Zhang H., He N., Wang J., Liu Y. (2020). Radiosensitization effect of AGuIX, a gadolinium-based nanoparticle, in nonsmall cell lung cancer. ACS Appl. Mater. Interfaces.

[117] Tian J., Wei X., Zhang W., Xu A. (2020). Effects of selenium nanoparticles combined with radiotherapy on lung cancer cells. Front. Bioeng. Biotechnol..

[118] Li Y., Yun K.-H., Lee H., Goh S.-H., Suh Y.-G., Choi Y. (2019). Porous platinum nanoparticles as a high-Z and oxygen generating nanozyme for enhanced radiotherapy in vivo. Biomaterials.

[119] Werner M.E., Cummings N.D., Sethi M., Wang E.C., Sukumar R., Moore D.T., Wang A.Z. (2013). Preclinical evaluation of Genexol-PM, a nanoparticle formulation of paclitaxel, as a novel radiosensitizer for the treatment of non-small cell lung cancer. Int. J. Radiat. Oncol. Biol. Phys..

[120] Hu Y., Paris S., Barsoumian H., Abana C.O., He K., Wasley M., Younes A.I., Masrorpour F., Chen D., Yang L. (2021). Radiation therapy enhanced by NBTXR3 nanoparticles overcomes anti-PD1 resistance and evokes abscopal effects. Int. J. Radiat. Oncol. Biol. Phys..

[121] Gao S., Zhang W., Wang R., Hopkins S.P., Spagnoli J.C., Racin M., Bai L., Li L., Jiang W., Yang X. (2020). Nanoparticles encapsulating nitrosylated maytansine to enhance radiation therapy. ACS Nano.

[122] Mokwena M.G., Kruger C.A., Ivan M.-T., Heidi A. (2018). A review of nanoparticle photosensitizer drug delivery uptake systems for photodynamic treatment of lung cancer. Photodiagnosis Photodyn. Ther..

[123] Pallavi P., Sharmiladevi P., Haribabu V., Girigoswami K., Girigoswami A. (2022). A Nano Approach to Formulate Photosensitizers for Photodynamic Therapy. Curr. Nanosci..

[124] Chen M.-H., Hanagata N., Ikoma T., Huang J.-Y., Li K.-Y., Lin C.-P., Lin F.-H. (2016). Hafnium-doped hydroxyapatite nanoparticles with ionizing radiation for lung cancer treatment. Acta Biomater..

[125] Ujiie H., Ding L., Fan R., Kato T., Lee D., Fujino K., Kinoshita T., Lee C.Y., Waddell T.K., Keshavjee S. (2019). Porphyrin–high-density lipoprotein: A novel photosensitizing nanoparticle for lung cancer therapy. Ann. Thorac. Surg..

[126] Yan L., Shen J., Wang J., Yang X., Dong S., Lu S. (2020). Nanoparticle-based drug delivery system: A patient-friendly chemotherapy for oncology. Dose-Response.

[127] Wang H., Yu J., Lu X., He X. (2016). Nanoparticle systems reduce systemic toxicity in cancer treatment. Nanomedicine.

[128] Sharmiladevi P., Girigoswami K., Haribabu V., Girigoswami A. (2021). Nano-enabled theranostics for cancer. Mater. Adv..

[129] Amsaveni G., Farook A.S., Haribabu V., Murugesan R., Girigoswami A. (2013). Engineered multifunctional nanoparticles for DLA cancer cells targeting, sorting, MR imaging and drug delivery. Adv. Sci. Eng. Med..

[130] Dracham C.B., Shankar A., Madan R. (2018). Radiation induced secondary malignancies: A review article. Radiat. Oncol. J..

[131] Han C., Wu Y., Kang K., Wang Z., Liu Z., Zhang F. (2021). Long-term radiation therapy-related risk of second primary malignancies in patients with lung cancer. J. Thorac. Dis..

[132] Wennstig A.-K., Wadsten C., Garmo H., Johansson M., Fredriksson I., Blomqvist C., Holmberg L., Nilsson G., Sund M. (2021). Risk of primary lung cancer after adjuvant radiotherapy in breast cancer—A large population-based study. NPJ Breast Cancer.

[133] Shruthi N., Samatha Jain M., Ganesan H., Banerjee A., Zhang H., Sun X.-F., Pathak S. (2023). Drug Repurposing in Cancer. Drug Repurposing for Emerging Infectious Diseases and Cancer.

